# Precision engineering of macrophage reprogramming with RNA interference-loaded lipid nanoparticles: a game-changer in cancer immunotherapy

**DOI:** 10.1007/s13346-025-01970-1

**Published:** 2025-09-18

**Authors:** Sezen Gül, Juliette Vergnaud, François Fay, Elias Fattal

**Affiliations:** Université Paris-Saclay, CNRS, Institut Galien Paris-Saclay, Orsay, 91400 France

**Keywords:** Tumor-associated macrophages, Reprogramming, Lipid nanoparticles, RNA interference

## Abstract

Tumor-associated macrophages (TAMs) represent solid tumors’ most prevalent immune cell subset. These cells primarily adopt an immunosuppressive phenotype in the tumor microenvironment, promoting tumor initiation and progression. Their ability to shift between distinct activation states identifies TAMs as ideal targets for cancer treatment. Consequently, reprogramming TAMs from an immunosuppressive to an immunostimulatory state has emerged as a promising therapeutic approach to fight cancer. RNA interference has gained significant attention as a therapeutic modality due to its potential to selectively inhibit the expression of one or several critical proteins for the pro-tumorous activities of TAMs. However, the efficiency of RNA interference is limited by its susceptibility to nuclease degradation, rapid clearance from the body, and poor cellular uptake. These limitations necessitate the development of delivery systems to enhance their therapeutic potential. Among the nanocarriers we discuss in this review, lipid nanoparticles (LNPs) have been widely recognized as the most effective for siRNA or miRNA, providing stability, high gene silencing efficiency, and biocompatibility. The clinical application of LNPs has been further advanced by recent progress in microfluidics, enabling reproducible and scalable production of LNPs with high encapsulation efficiency. The increasing number of preclinical studies shows the growing interest in cancer immunotherapy using RNA interference-LNPs. In this review, we summarize the current knowledge on macrophage biology and its role in cancer, explore advancements in RNA interference-LNP technology, review ongoing research efforts, and discuss key translational challenges that must be addressed for the clinical success of RNA interference-LNP-based macrophage reprogramming.

**Graphical abstract:**

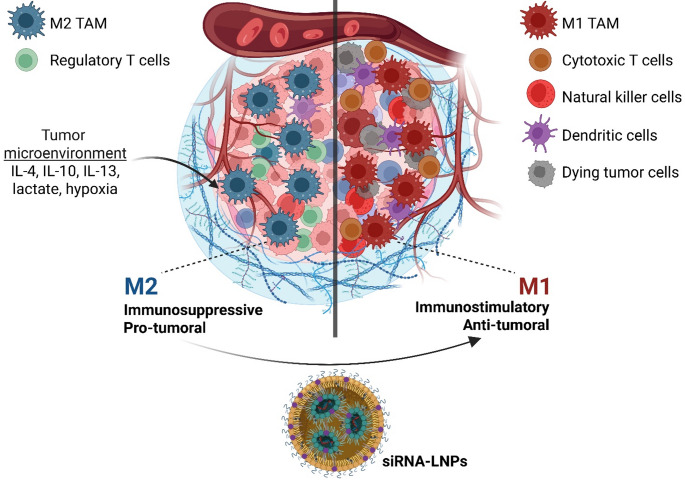

## Introduction

The tumor microenvironment (TME) is composed of a broad array of cellular components such as fibroblasts, endothelial cells, stromal cells, immune cells, and cancerous cells, along with non-cellular elements such as extracellular matrix (ECM), signaling molecules, and extracellular vesicles [1]. In this dynamic environment, macrophages are particularly abundant and can adapt their function in response to local signals [2]. In many solid tumors, tumor-associated macrophages (TAMs) are predominantly polarized toward a pro-tumoral phenotype, supporting tumor progression by suppressing immune responses, promoting angiogenesis, and facilitating tissue remodeling [3]. The functional polarizability of macrophages provides an opportunity to develop targeted therapeutic strategies.

Recent studies have focused on reprogramming macrophages from a pro-tumoral to an anti-tumoral state as a promising strategy to treat cancer [4–6]. Various repolarization approaches have been investigated, including small-molecule drugs [7, 8] and monoclonal antibodies [9, 10]. However, due to their non-specificity, these strategies are often accompanied by potential systemic toxicity and other adverse effects. In this context, RNA-interference-based therapeutics have been identified as powerful tools to selectively modulate gene expression in macrophages and, thereby, their activity [11, 12]. However, efficient nucleic acid delivery to TAMs remains a significant challenge [13, 14].

Lipid nanoparticles (LNPs) have appeared as a leading delivery platform for RNAs, overcoming several barriers, including RNA degradation, poor cellular uptake, and limited endosomal escape [13]. The clinical success of LNPs in delivering siRNA (Patisiran, Alnylam Pharmaceuticals [12]) and mRNA (mRNA-1273, Moderna; BNT162b2, Pfizer-BioNTech [15]) has further proven the effectiveness of this platform in various therapeutic applications. Consequently, RNA-loaded LNPs hold great promise for precisely modulating TAM activity, paving the way for the next generation of cancer immunotherapies [13]. However, several challenges must be overcome for their translation to clinics, including efficient targeting of macrophages in the TME, optimization of intracellular delivery, and minimization of off-target effects [13, 16].

This literature review provides a comprehensive overview of macrophage reprogramming using RNA interference-LNPs. We start by describing the biology of macrophages, their role in cancer, and therapeutic reprogramming strategies. Then, we introduce the mechanism of siRNA and miRNA-based gene silencing, LNPs as RNA delivery systems, and key aspects that should be considered to optimize the efficacy of RNA interference-LNPs. Finally, we highlight the state-of-the-art applications of RNA interference-LNPs in TAM reprogramming, along with critical challenges and future perspectives that will drive the clinical translation of this approach. We hope to advance theoretical understanding and the application of RNA interference-LNP-based TAM reprogramming in cancer therapy by addressing these key factors.

## Macrophages in health and disease

Macrophages are highly specialized immune cells that arise from two distinct developmental pathways. While tissue-resident macrophages derive mainly from embryonic origins, bone marrow-derived macrophages (BMDMs) originate from circulating monocytes produced in the bone marrow [17, 18]. Tissue-resident macrophages (e.g., microglia, Kupffer cells, Langerhans cells, and alveolar macrophages) are capable of self-renewal and have a longer life span than BMDMs [4, 17–20]. Belonging to the mononuclear phagocyte system (MPS) [17], these cells play a vital role in providing host defense against infections and maintaining tissue homeostasis [4, 21]. They are crucial modulators and effector cells in inflammatory responses [22].

Macrophages have long been known for their ability to shift between distinct functional states based on environmental cues [23, 24]. This remarkable plasticity enables them to adapt to the specific needs of the microenvironment but also underlies their involvement in the development and progression of various diseases, including cancer, infection, chronic inflammation, and autoimmune diseases.

### Macrophage polarization: M1 vs. M2 phenotypes

The polarization into various activation states operates on a spectrum, and macrophages can take on intermediate phenotypes depending on the specific stimuli they encounter within the local tissue environment. Despite this complexity, macrophage polarization is generally categorized into two major groups: the pro-inflammatory M1 and the anti-inflammatory M2 states [2, 4, 17, 25–27].

**Fig. 1:**
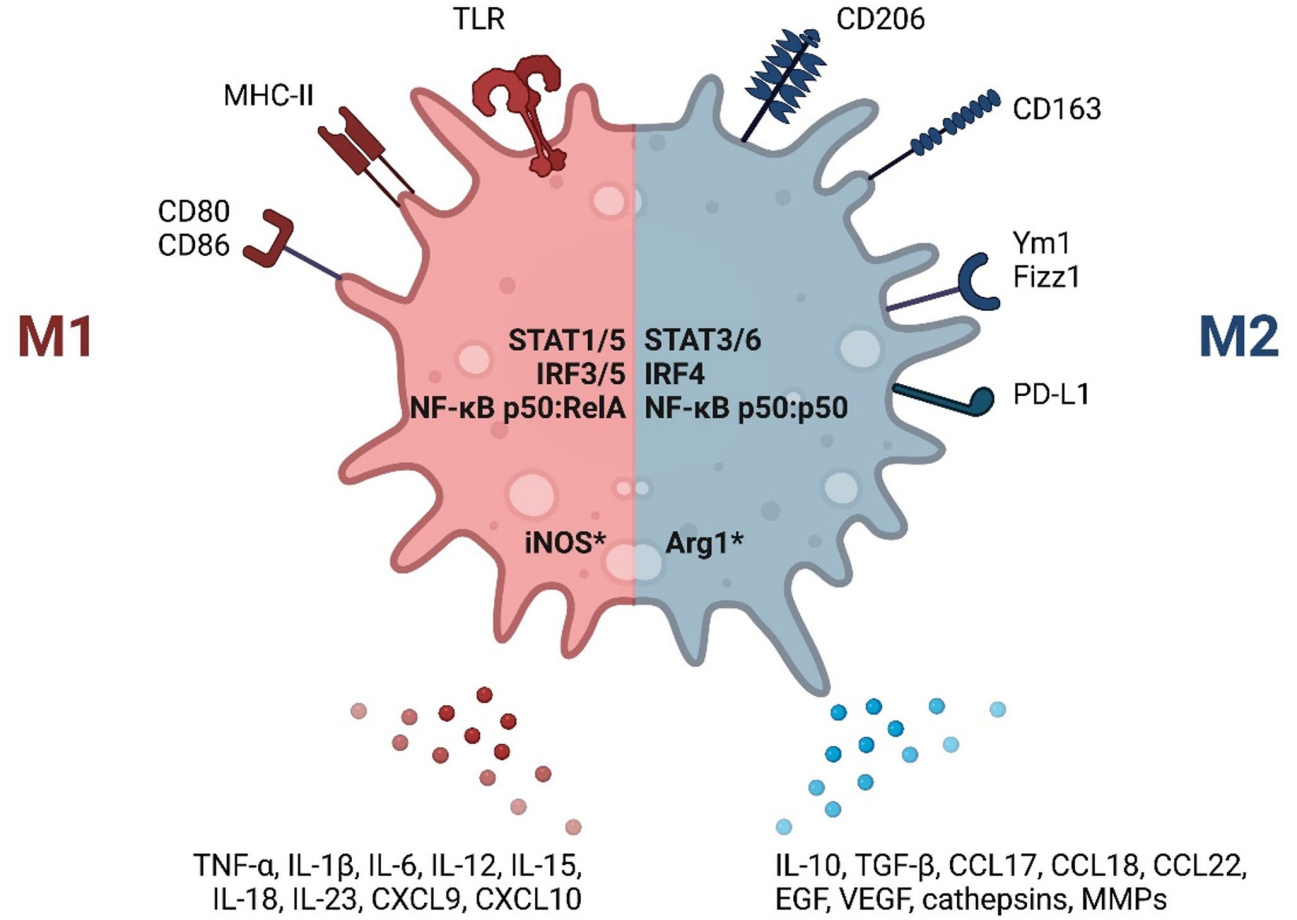
Macrophage polarization and marker expression in different types of activated macrophages: M1 (left) vs. M2 (right). Adapted from [22, 23, 29, 30]

Polarization of macrophages into the M1 state is driven by pathogen-associated molecular patterns (e.g., LPS from gram-negative bacteria) or pro-inflammatory cytokines (e.g., IFN-γ). It is essential for immune defense [21, 27]. M1 macrophages secrete high levels of pro-inflammatory cytokines, including TNF-α, IL-1β, IL-6, and IL-12, crucial for recruiting and activating T cells, natural killer cells, and dendritic cells. They also enhance the adaptive immune response by presenting antigens to T cells and can kill tumor cells or pathogens directly through phagocytosis. Furthermore, their production of nitric oxide (NO) and reactive oxygen species (ROS) may provoke apoptosis in neighboring cells [4, 23]. Conversely, M2 activation is typically induced by anti-inflammatory cytokines such as IL-4, IL-13, and IL-10 [27, 28]. These cells promote tissue repair, angiogenesis, ECM remodeling, and the resolution of inflammation through the secretion of anti-inflammatory cytokines, including IL-10 and TGF-β, and growth factors [4, 23, 26, 28]. The activation state of macrophages can be determined based on their distinct cytokine or chemokine secretion profiles, cell surface receptors, transcriptional regulators, and other mediators (Fig. 1).

The M1/M2 classification represents the extremes of a functional spectrum, and macrophages undergo a dynamic transition between these two states in response to environmental signals. Importantly, dysregulation of this balance has been associated with various diseases. One example is cancer, where macrophages are often skewed towards a tumor-supportive M2-like phenotype.

### Role of macrophages in cancer resistance

TAMs represent solid tumors’ most abundant immune population, pivotal in shaping the TME [2, 31]. These immune cells originate from tissue-resident macrophages or circulating monocytes, which are recruited to the tumor site and differentiate into macrophages [2]. Specific chemokines (e.g., CCL2, CCL3, CCL5, CCL15, CCL26, CXCL8, CXCL12), cytokines (e.g., CSF-1, GM-CSF), and complement proteins (e.g., C5) are crucial in the recruitment of macrophages to the tumor site [1–3, 32].

**Fig. 2:**
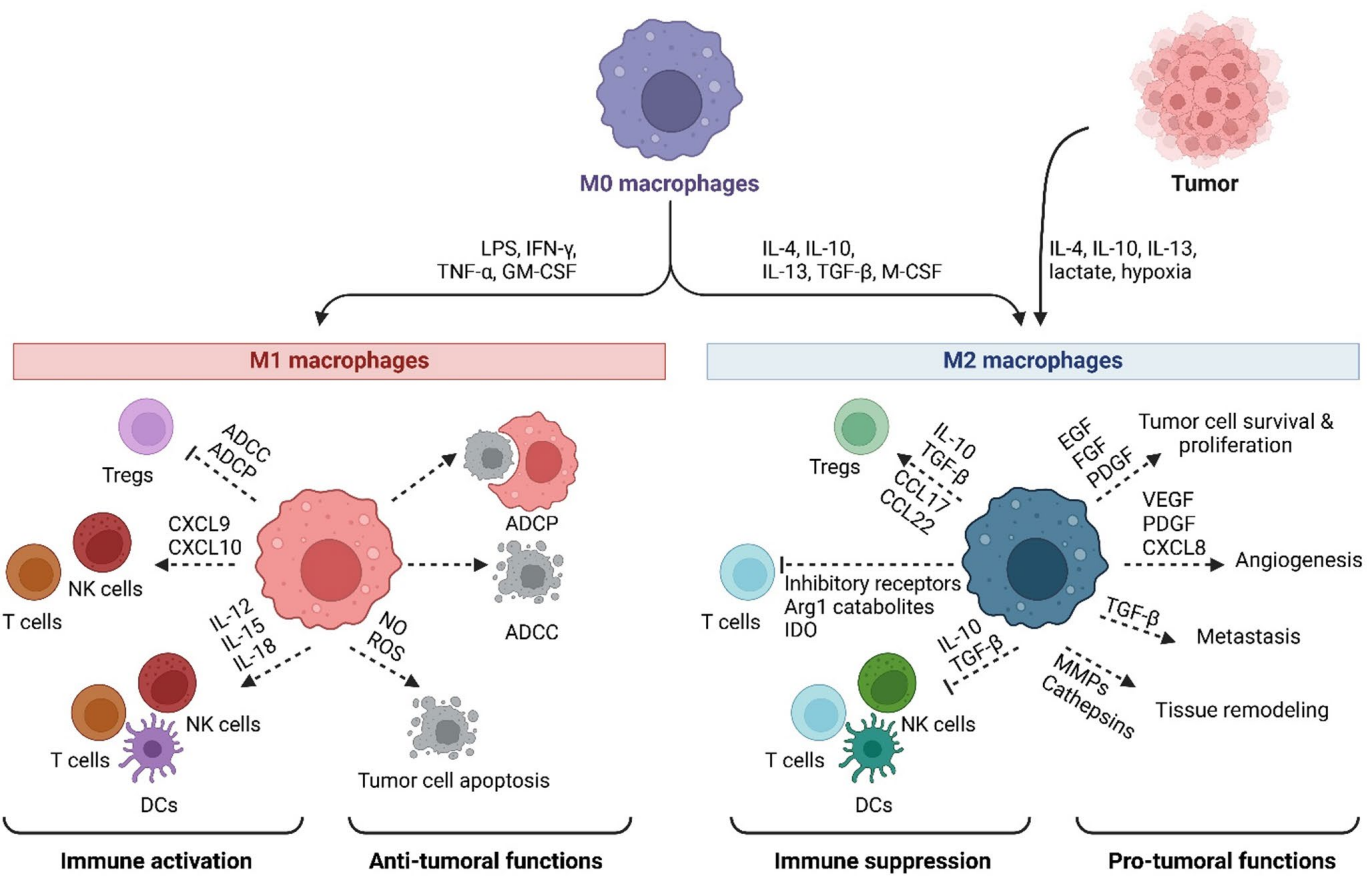
Tumor-promoting and suppressing roles of TAMs. TAMs can be polarized into two states with distinct functions depending on external stimuli. ADCC, antibody-dependent cellular cytotoxicity; ADCP, antibody-dependent cellular phagocytosis; DCs, dendritic cells; NK cells, natural killer cells. Adapted from [2, 30]

Two distinct polarization phenotypes, M1 and M2, can be identified within the TME. While M1 macrophages contribute to inflammatory and anti-tumor immune responses, M2 macrophages participate in tumor cell proliferation, survival, and metastasis, as well as angiogenesis and immunosuppression (Fig. 2). Consequently, macrophages play a dual role in tumor progression, where their quantity and polarization state affect patient survival, with the M2-like phenotype being strongly linked to poor clinical outcomes in most solid tumors [27, 33–40].

The TME is typically characterized by hypoxia, low pH, and elevated interstitial fluid pressure, which are primarily caused by the abnormal formation of new blood vessels [31]. In the TME, several functional determinants, including hypoxia, tumor-derived lactate, IL-4, IL-13, and IL-10, collectively induce the differentiation of macrophages into the pro-tumor M2 phenotype [3]. These M2-like macrophages enhance immunosuppression through the secretion of cytokines such as TGF-β and IL-10 [6, 27, 41]. M2-like macrophages also inhibit the activation and function of effector immune cells by expressing immune checkpoint molecules, including PD-L1, TIM-3, and VISTA [4]. They suppress the activity of cytotoxic (CD8+) and helper (CD4+) T cells via the action of various molecules, including IL-10, TGF-β, CCL2, cathepsin K, COX-2, and matrix metalloproteinases (MMPs), while promoting the recruitment of regulatory T cells (Tregs) [2, 41]. Moreover, these cells promote angiogenesis by producing factors like VEGF, PDGF, TGF-β, CXCL8, MMP9, TIE2, and ANG2, thereby facilitating oxygen and nutrient delivery to the tumor [2]. The secretion of molecules such as MMPs, serine proteases, and cathepsins by these cells also contributes to tumor invasion and metastasis by degrading the ECM and disrupting cell-cell/cell-ECM interactions [2, 42]. Additionally, TAMs support cancer stem cells’ survival, self-renewal, and tumorigenicity through factors such as IL-6 and TGF-β [43, 44]. Finally, these cells are critical in developing resistance to conventional cancer treatments, including chemotherapy, radiotherapy, and immunotherapies [2, 27, 41, 45].

Despite their predominantly pro-tumoral role within the TME, macrophages can also progress into an anti-tumoral M1-like phenotype. M1-like macrophages have been shown to hinder cancer progression by inducing tumor cell apoptosis by producing ROS and NO. They also possess the potential to phagocytose tumor cells and present tumor-specific antigens for adaptive antitumor immunity [2, 30]. This functional duality offers a groundbreaking therapeutic opportunity in cancer treatment, where TAMs can be reprogrammed from tumor supporters into potent tumor fighters.

## Molecular targets and therapeutic approaches for macrophage reprogramming

Reprogramming TAMs from the immunosuppressive M2 to the immunostimulatory M1 phenotype holds great promise for treating cancer. Restoring the anti-tumor function of these cells can stimulate a robust immune response to promote tumor clearance [4–6].

Although several TAM-related therapeutic strategies, such as the inhibition of macrophage recruitment into tumors and macrophage survival, are currently under investigation, TAM reprogramming by molecular targeting stands out as the most promising approach [30, 41, 46]. This strategy may suppress the pro-tumoral functions of TAMs and activate their anti-tumoral activities. M2-to-M1 repolarization can enhance the efficacy of other anti-tumor therapies, including chemotherapy and radiotherapy, as well as immunotherapies currently in clinical use, including immune checkpoint inhibitors and chimeric antigen receptor (CAR) T cells [2, 46].

**Fig. 3:**
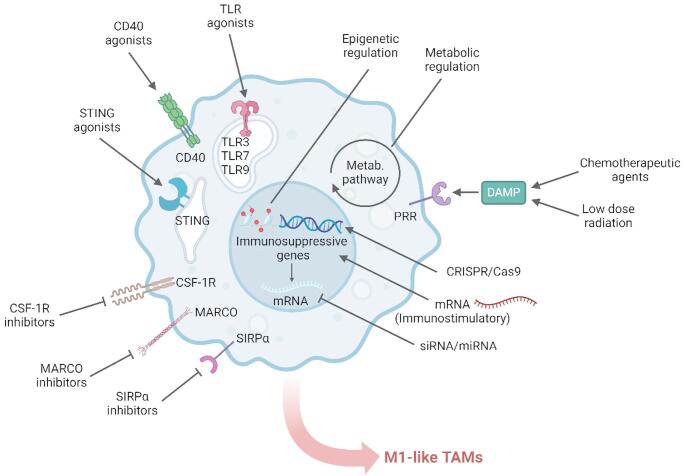
TAM reprogramming strategies and targets: M2-to-M1 polarization can be induced by activating immunostimulatory mediators or inhibiting immunosuppressive ones. Adapted from [2]

Various strategies have been devised to induce M2-to-M1 reprogramming of TAMs (Fig. 3). To begin with, the activation of toll-like receptors (TLR) has been shown to drive macrophages toward an M1-like phenotype. TLRs are key pattern recognition receptors expressed in TAMs, enabling these cells to detect damage-associated molecular patterns (DAMPs) and initiate immune responses [7]. The activation of toll-like receptors (TLR) has been addressed using a TLR7 agonist (Imiquimod), approved by the FDA, illustrating the possibility of using these types of molecules in anti-tumor therapy [2, 7, 29]. Other TLR activators have been applied, including poly(I: C) (TLR3 agonist) [47–49] and synthetic unmethylated cytosine-guanine (CpG) oligodeoxynucleotides (TLR9 agonist) [50–52], which have also been shown to repolarize TAMs and enhance immunostimulatory activity. Nevertheless, the systemic administration of free TLR agonists, mostly small molecules, has been limited due to the side effects of a systemic interferon reaction [53]. However, it has stimulated the development of local delivery systems and nanoparticulate drug carriers with little clinical success [54–56].

Another similar approach involves using the stimulator of interferon genes (STING) agonists. STING, a cytoplasmic protein in the endoplasmic reticulum, is crucial for detecting cytosolic DNA caused by cellular damage. Its activation in TAMs stimulates IRF3, type I interferon genes, and pro-inflammatory cytokines [54, 55]. At least twenty agonists of STING, most of them small molecules, have been clinically evaluated in Phase 1 and 2 trials. The first trials, which involved the intratumoral administration of synthetic cyclic dinucleotides (e.g., ADU-S100, MK-1454), demonstrated limited cell penetration, susceptibility to enzymatic degradation, and insufficient efficacy [56–58]. Consequently, liposomes loaded with STING agonist cGAMP have yielded more promising results in murine models of breast cancer and lung metastasis [59, 60]. Other approaches using nanoparticles or exosomes are under investigation [61, 62].

Inducing CD40 activation using monoclonal antibodies (mAbs) is another effective strategy for stimulating the antitumor functions of TAMs [9]. CD40 is a protein belonging to the TNF receptor family that is expressed on TAMs [63], and its stimulation can increase the release of TNF-α and NO from TAMs, enhancing CD8 + T cell activation [29, 63]. CD40 agonists are currently being assessed in clinical trials in combination with chemotherapy [64]. The combination of antibodies targeting CD40 and CSF-1R has been shown to effectively treat preclinical tumor models that were not responsive to immune checkpoint inhibitors by impairing the recruitment of new TAMs towards immunosuppressive cells, reprogramming TAMs, and activating cytotoxic T cells, thus unleashing potent antitumor immunity [65, 66]. Various anti-CD40 mAbs have been clinically evaluated alongside immune checkpoint inhibitors, chemotherapy, and other targeted therapies [67]. However, several of those have been discontinued due to modest efficacy and dose-limiting toxicities such as cytokine release syndrome, liver toxicity, and enhanced angiogenesis [68]. A similar mechanism of action has been observed for anti-MARCO mAbs in murine models of breast, colon, and melanoma cancers, promoting the anti-tumoral functions of macrophages in tumors [10]. These antibodies target the pattern-recognition scavenger receptor MARCO, which is overexpressed in TAMs and associated with a poor prognosis in breast, lung, and liver cancers [69–71]. Moreover, bispecific antibodies targeting angiopoietin-2 (Ang-2) and vascular endothelial growth factor (VEGF) have been shown to reprogram TAMs and delay tumor growth in murine glioma models. Combining Ang-2/VEGF bispecific antibodies with 5-fluorouracil (5-FU) and irinotecan in colorectal cancer, or with temozolomide in glioma, has shown significant benefits compared to anti-VEGF combined with chemotherapy [72, 73].

Another interesting approach has focused on using mAbs to reprogram TAMs into antitumor effectors by manipulating the CD47-signal regulatory protein alpha (SIRPα) axis. SIRPα, which is expressed on the surface of phagocytic cells such as macrophages, interacts with CD47 which is overexpressed by cancer cells. This interaction inhibits phagocytosis, acting as a “don’t eat me” signal for tissue homeostasis [74]. In preclinical cancer models, the pharmacological inhibition of CD47 restores macrophages’ ability to phagocytose and kill tumor cells [75–77]. Although antibodies able to inhibit SIRPα showed satisfactory antitumor activity in lung cancer models, their effects are limited in time [78]. Self-assembling SIRPα-blocking antibodies with CSF-1R inhibitors have successfully reprogrammed TAMs into M1 antitumor effectors. This combined therapy activates antitumor macrophages by hindering CD47-SIRPα ligation while inhibiting the recruitment of new TAMs [79, 80]. Clinical trials using anti-CD47 mAbs or CD47-Fc fusion proteins are ongoing for the treatment of hematological cancers or refractory solid tumors, either alone or in combination with anti-PD-1 therapy or anti-CD20 (Rituximab^®^) to target B cells [81].

Macrophage polarization is also associated with alterations in cellular metabolism, as indicated by their differential metabolization of glucose, lipids, amino acids, oxygen, and iron [2]. While M1 macrophages predominantly depend on glycolysis, M2 macrophages utilize oxidative phosphorylation [82, 83]. Fatty acid oxidation is crucial for M2 polarization [84]. The L-Arginine catabolic pathway leads to NO and L-citruline production by iNOS in the M1 state, whereas Arg1 produces ornithine and urea in the M2 state. Thus, increased expression of Arg1 is linked to immunosuppressive activities [85]. The overexpression of indoleamine 2,3-dioxygenase (IDO), an enzyme catabolizing tryptophan into kynurenine, has been associated with the induction of M2-like TAMs [86, 87]. Furthermore, hypoxia induces TAMs polarization toward an M2-like phenotype [88]. Regarding iron metabolism, the M2 state is characterized by an elevated iron release and limited iron storage compared to the M1 [89]. Various therapeutic approaches mediating glycolysis, arginase, IDO, hypoxia, or iron metabolism in macrophages have been investigated for their potential in reprogramming, with promising results. Finally, it has been demonstrated that traditional chemotherapeutic agents (e.g., doxorubicin) [3], oncolytic viruses [90], and low doses of radiation [91, 92] shift macrophages towards an M1-like state by triggering the release of DAMPs by cancer cells, which stimulate the pattern recognition receptors (PRRs) on TAMs [2].

Epigenetic regulation of macrophages has been investigated through the mediation of histone deacetylases [93, 94], histone methyltransferases, or histone demethylases [94], with several current clinical trials evaluating the potential of combining epigenetic modifications with immune checkpoint inhibitors [95]. Furthermore, various targets to modulate gene transcription of TAMs toward the M1-like subtype have been addressed using nucleic acids such as siRNA, microRNA (miRNA) [2]. Their delivery will be discussed in more detail later in this review, but they were designed to inhibit mediators, including STAT6 [96], IκBα [97, 98], and CSF-1R [99–101]. Upregulation of miRNAs such as miR-125a [102], miR-125b [103], and miR155 [104] induced successful TAM repolarization toward the M1 phenotype and enhancement of antitumor immune response. Moreover, mRNAs encoding OX40L [105], CD80, and CD86 [106], administered intratumorally, could overcome the local immunosuppressive environment of a tumor. Finally, the CRISPR/Cas9 system and lentiviral vectors have been employed to edit the genome of TAMs directly [2]. However, these gene-editing techniques still pose some challenges in clinical settings, particularly regarding their safety.

A recent review focusing on the clinical landscape of macrophage-reprogramming cancer immunotherapies has stressed that, despite the large number of targets and clinical trials, only a few exceeded Phase 2 [107]. Common reasons for this failure include toxicity due to the indiscriminate activation of macrophages (e.g., TLR agonists), leading to severe adverse effects [108], and the insufficient sustained inhibition of signaling pathways involved in macrophage reprogramming (e.g., STING) [109, 110]. One of the reasons could be that most of those strategies are based on intracellular targets that are difficult to reach in a clinical setting [111]. Additionally, despite advances in the development of humanized and fully human antibodies, there is still a risk of patients developing anti-drug antibodies (ADAs) against the therapeutic antibody. ADAs can alter their pharmacokinetics, neutralize the antibody’s effect, and even lead to infusion reactions [112].

Compared to antibodies and small molecule drugs, which exert their therapeutic action by binding to particular proteins and require the identification of highly active and specific target proteins related to diseases, siRNA and miRNA provide a more promising therapeutic approach regarding their easier design and shorter development period, as well as the possibility to theoretically knock down any disease-associated genes [113–116].

## RNA interference and administration hurdles

MicroRNAs (miRNAs) and small interfering RNAs (siRNAs) are small RNA molecules important for gene regulation through RNA interference (RNAi). While siRNAs are designed to block one target gene, miRNAs can target multiple genes [118]. New strategies have been developed focusing on miRNA as therapeutic candidates (in the form of miRNA mimics) or as molecular targets using antimicro-RNAs (Antagomirs) as therapeutic moieties [117, 118].

**Fig. 4:**
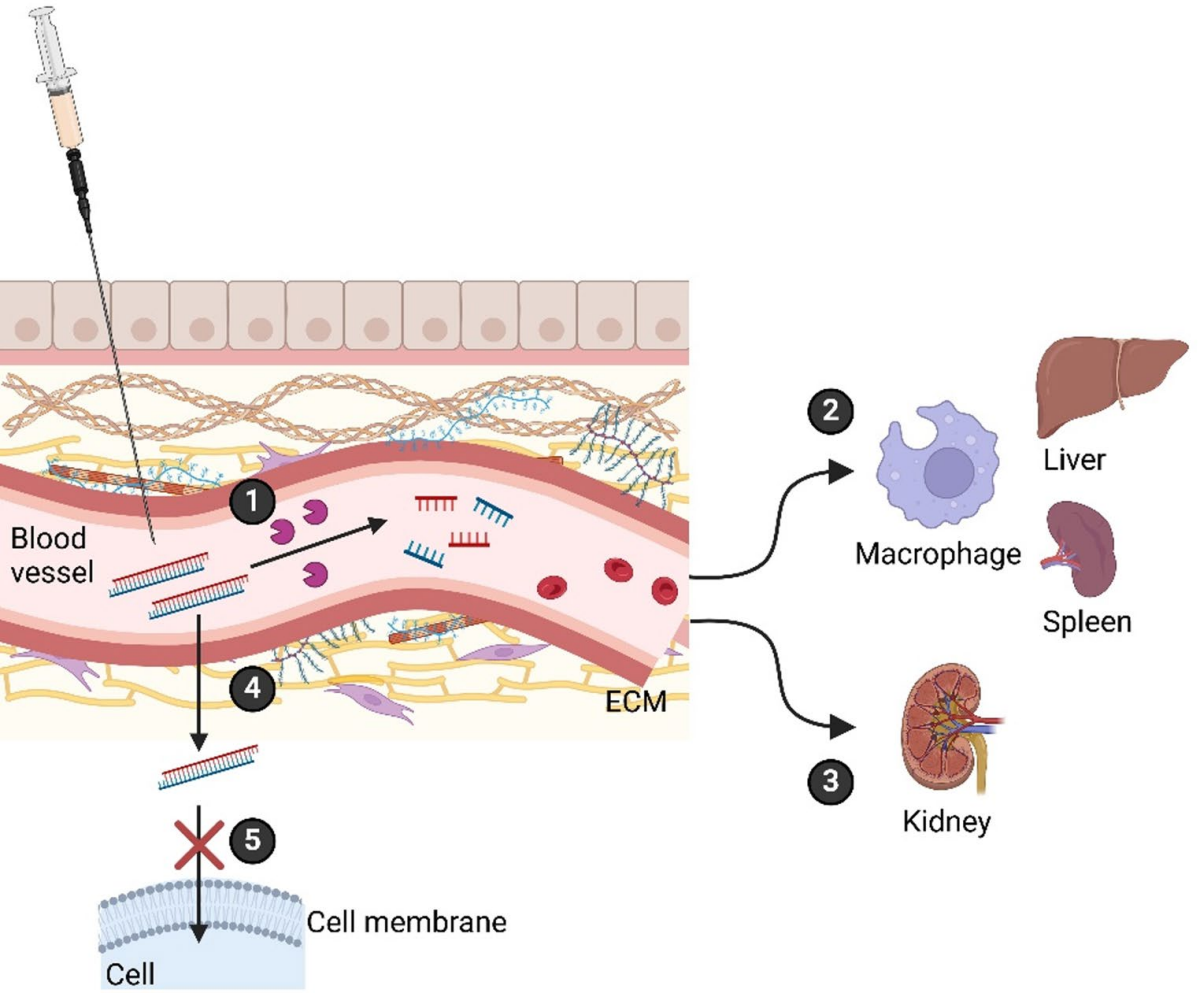
Physiological barriers to systemic siRNA delivery: Various in vivo barriers to free siRNA delivery: (1) degradation by endonucleases, (2) uptake by the MPS, (3) renal clearance, (4) extravasation and transport across the interstitial space, and (5) transport across the cell membrane. Adapted from [121, 124]

Depending on the target tissue and the administration route, there are various barriers to the delivery of free therapeutic RNA to its site of action. Following intravenous injection, the RNA formulation must overcome a variety of in vivo barriers: enzymatic degradation by serum or tissue nucleases, renal clearance, uptake by the MPS, extravasation and transport across the interstitial space, transport across the cell plasma membrane, endosomal entrapment, and lysosomal degradation (Fig. 4) [119–122]. Moreover, another barrier consists of their degradation by endogenous nucleases. RNA are also quickly cleared by glomerular filtration in the kidneys. Another biological barrier is the uptake by the MPS, which is a population of phagocytic cells that effectively eliminate pathogens, cellular debris, and foreign substances from the blood circulation and tissues. After systemic administration, higher concentrations of RNA are observed in the liver and spleen due to high blood flow to these organs, their discontinuous vessels, and numerous tissue macrophages. The crossing of cell membranes poses another challenge for free RNA delivery, given that it is a hydrophilic molecule with a net negative charge [121, 122]. It is also noteworthy that high levels of RNA have the potential to activate the innate immune system via the TLR pathways [120, 122]. Subsequently, the half-life of unmodified and free RNA in serum has been reported to range from several minutes to 1 h [122], with the majority exhibiting a half-life of approximately 10 min [121, 123].

Different strategies have been proposed to address the abovementioned challenges. The first approach involves altering RNA through chemical modifications, and the second focuses on developing nanoparticle-based RNA delivery systems. Regarding the initial strategy, RNA, particularly siRNA, can be chemically modified on various groups, such as bases, sugars, or phosphate groups, to enhance its in vivo stability by increasing its resistance to nucleases and avoiding its immune recognition. Typical modifications include the conversion of the phosphodiester (PO_4_) group into the phosphorotioate (PS) group and the replacement of the 2’-hydroxyl group of the ribose ring by various groups, including 2’-O-methyl, 2’-O-methoxyethyl, and 2’-fluoro. Those chemical modifications should be designed and tested so as not to affect gene silencing adversely. As for the second strategy, scientists have developed a variety of nanocarriers, including lipid-based and polymer-based systems, which can protect the siRNA from degradation, facilitate its cellular uptake, and deliver it to target sites [116, 122].

## Non-lipid nanoparticles for the delivery of RNA interference in macrophage reprogramming

This review focuses on using lipid nanoparticles to deliver RNA interference (siRNA, miRNA) for reprogramming macrophages. However, other types of M2-targeted or non-targeted nanoparticles have also been developed to deliver siRNA or miRNA. These nanoparticle platforms vary significantly in composition, for example, some utilize an amphiphilic cationic β-cyclodextrin (CD) incorporating CSF-1R siRNA and a targeting peptide (M2pep) [125]. Others are based on cationic polymers, such as poly(ethylenimine), that can be coupled with hyaluronic acid to target the CD44 receptor [103, 126].

Indeed, silencing STAT6 was first carried out using polyethyleneimine or polypropylenimine nanoparticles [96]. JAK/STAT pathway plays a central role in regulating macrophage activation states. The IL-4- or IL-13-induced activation of STAT6 is crucial in expressing M2-associated genes such as Arg1, KLF4, PPARγ, CD206, and Fizz1. Moreover, STAT3, which IL-10 activates, is also associated with the M2 state by activating genes such as IL-10, TGF-β1, CD206, and Arg1 [127]. In the study by Walther et al. [96], the researchers demonstrated that siRNA-mediated silencing of STAT6 using the cationic polymers is a promising approach to trigger M2-to-M1 polarization in vitro, resulting in enhanced tumor cell phagocytosis in a co-culture model.

The classical and alternative pathways of NF-κB signaling also function as critical regulators of TAM polarization [128, 129]. Activation of the NF-κB pathway has been linked to both anti-tumor and pro-tumor functions in TAMs [97, 129, 130]. Although the mechanism by which NF-κB modulates TAM polarization is still an ongoing area of research, several studies have recently demonstrated that activation of the classical NF-κB pathway induces an M1-like state in macrophages [97, 131]. Therefore, silencing molecules associated with NF-κB p50:RelA inhibition, such as IκBα, can promote pro-inflammatory signaling and shift macrophages toward an anti-tumor M1 phenotype [97, 131]. In a study by Ortega et al. [97], the researchers employed mannosylated methacrylate-based nanoparticles to deliver siRNA against IκBα to activate the NF-κB pathway and boost anti-tumor functions in macrophages, yielding promising results. In a separate study, Glass et al. [98] have also shown that the treatment with this mannose-functionalized polymeric nanoparticles loaded with siRNA targeting IκBα repolarized macrophages toward a pro-inflammatory phenotype and significantly decreased tumor burden in mouse models of ovarian cancer [131].

Another polarization modulator is CSF-1R, which has been implicated in the infiltration, survival, and immunosuppressive function of TAMs [132, 133]. Sun et al. [125] demonstrated that targeting the CSF-1R with siRNA through the use of cyclodextrin-based nanoparticles decorated with M2 macrophage-targeting peptide promoted the immunostimulatory activities of macrophages in vivo, enhanced the helper and cytotoxic T cell infiltration in the TME, and triggered cancer cell apoptosis in a mouse model of prostate cancer.

Treatment strategies simultaneously targeting TAMs through multiple pathways are an elegant option to address the tumor’s complex nature. Combination strategies can overcome therapeutic resistance and enhance immune responses, improving treatment efficacy compared to single-agent therapies [6, 101]. Pan et al. [134] illustrated that the combined administration of siRNAs against CSF-1R and signal-regulatory protein alpha (SIRPα) effectively reprograms IL-4-stimulated BMDMs towards an M1-like state, promoting macrophage-induced phagocytosis of cancer cells. Additionally, administering these nanoparticles to Lewis lung carcinoma-implanted mice resulted in a notable reduction in tumor size. Similarly, Cheng et al. [100] presented an innovative approach employing DNA tetrahedron nanostructures loaded with siRNA against CSF-1R and immune adjuvant CpG for pro-inflammatory macrophage reprogramming. The treatment induced apoptosis and necrosis in 4T1 tumor cells in vitro and inhibited tumor growth in 4T1 xenograft tumor-bearing mice. Moreover, the study of Li et al. [101] demonstrated that the treatment with nanomicelles that are actively targeted to M2 TAMs for the co-delivery of siRNA against CSF-1R and the PI3K-γ inhibitor NVP-BEZ 235 effectively remodeled the TME by increasing M1 polarization and decreasing myeloid-derived suppressor cell (MDSC) infiltration in the context of pancreatic cancer. In another study, Xiao et al. [6] showed that the M2-targeted co-delivery of siRNA against IKKβ and the STAT6 inhibitor AS1517499 by pH-responsive micelleplexes efficiently induced M2-to-M1 polarization in vivo and effectively attenuated tumor growth and metastasis of the 4T1 breast tumor in mice. Using CD44 targeting hyaluronic acid-poly(ethylenimine) (HA-PEI)-based nanoparticles encapsulating miR-125b, it was possible to transfect TAMs in lung tissues in a non-small cell lung cancer mouse model and to successfully repolarize them into the M1 phenotype [103].

## Lipid nanoparticles for the delivery of RNA interference in macrophage reprogramming

Compared to cationic nanoparticles, LNPs allow for higher encapsulation efficiency and greater stability in biological fluids. LNPs are much less toxic than cationic liposomes, which have a persistent positive charge that leads to higher cytotoxicity and instability in vivo due to non-specific interactions with serum proteins. Polymeric nanoparticles offer versatility in terms of material properties and controlled release. However, they often face challenges in achieving high encapsulation efficiency for nucleic acids, efficient endosomal escape, and favorable biocompatibility profiles compared to LNPs. Some polymeric systems also suffer from poor serum stability and can induce immunogenicity. One final advantage of LNPs is the reproducibility of manufacturing techniques, especially those using microfluidics, which allow precise control over particle size, uniformity, and encapsulation efficiency. This leads to highly reproducible batches. These scalable methods enable the large-scale production required for clinical trials and widespread therapeutic use. Importantly, as LNPs have already been clinically validated to deliver siRNA and mRNA for several applications [113] they have naturally attracted the widespread attention from both academia and industry.

### Composition influence on physico-chemical and biological properties of LNPs

LNPs have been developed as vehicles to overcome the challenges associated with RNA interference delivery by efficiently encapsulating RNA, shielding it from serum nucleases and immune system molecules, decreasing its renal clearance, and facilitating its transmembrane transport and endosomal escape [135]. Functionalizing the LNP surface with targeting moieties may also contribute to the selective delivery of RNA to specific cells, although tissue distribution should theoretically remain similar to non-targeted LNP [136]. This section provides an overview of the mechanism of LNP-mediated siRNA delivery and then emphasizes the critical role of lipid composition in optimizing RNA interference-LNP performance.

**Fig. 5:**
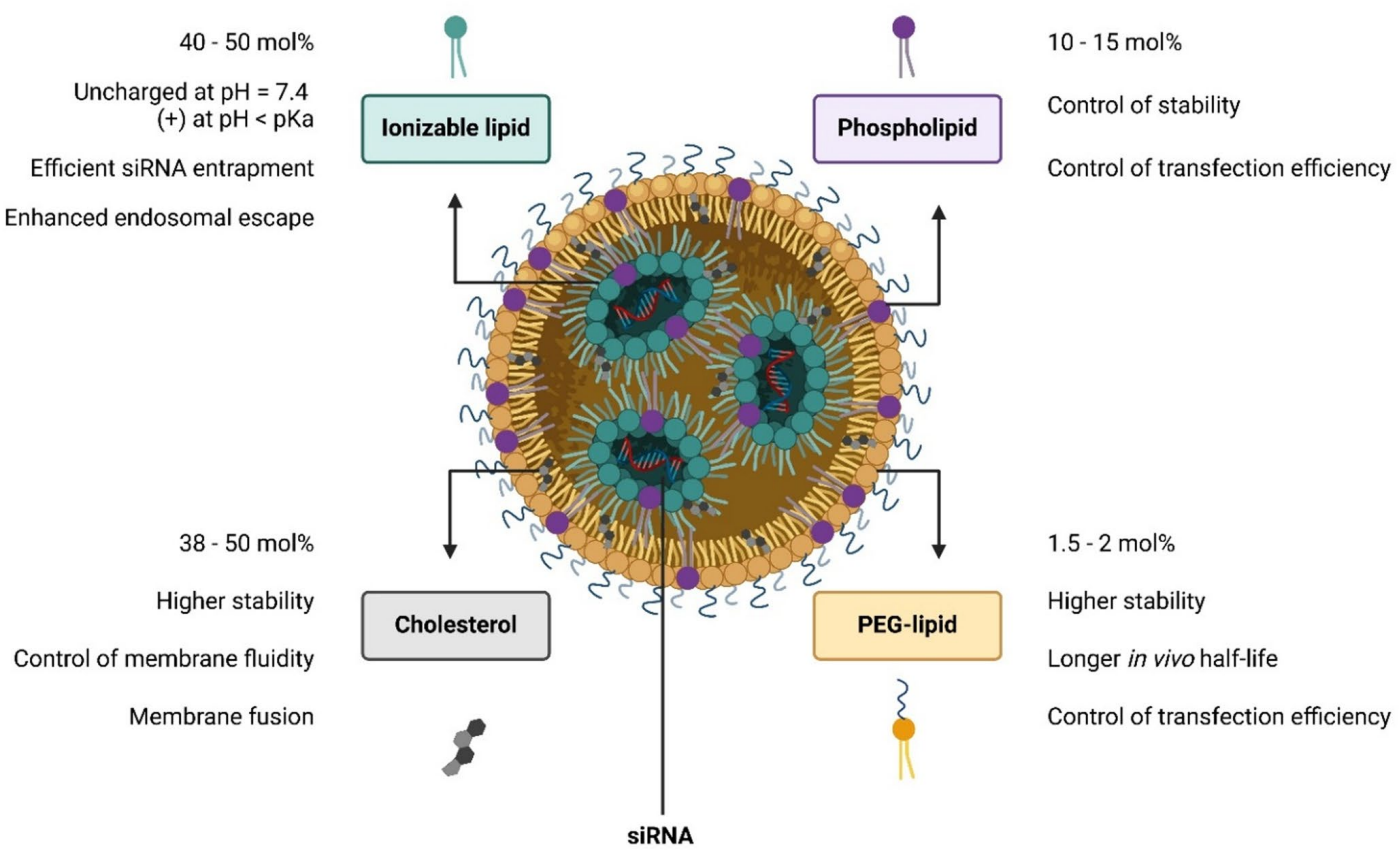
Schematic representation of RNA-LNP structure and its main components. Ionizable lipids, helper phospholipids, cholesterol, and PEG-lipids are illustrated with their associated characteristics. Adapted from [13]

The lipid composition impacts the therapeutic efficacy of siRNA-LNPs [16]. In general, LNPs consist of four main constituents (Fig. 5): an ionizable cationic lipid (40–50 mol%), a helper phospholipid (10–15 mol%), cholesterol (38–50 mol%), and polyethylene glycol (PEG) coupled to a lipid moiety, generally phospholipid (1.5–2 mol%) [13, 135, 137].

#### Ionizable lipids

The critical importance of ionizable cationic lipids in the formulation stems from the fact that they are relatively uncharged at the physiological pH of 7.4. In contrast, they become positively charged at pH values lower than their acid dissociation constant (pK_a_). This leads to efficient RNA encapsulation in LNPs [138]. During LNP production, which is carried out at acidic pH (pH$\:\sim$4), the protonation of ionizable lipids contributes to the encapsulation of anionic nucleic acids within nanoparticles as a result of electrostatic interactions, allowing high encapsulation efficiencies [16].

Ionizable lipids play an essential role in the intracellular trafficking of RNA after cell uptake. Cell uptake of nanoparticles primarily occurs through endocytosis, which can be classified into phagocytosis and pinocytosis. Phagocytosis is a process that occurs exclusively in professional phagocytes such as macrophages, monocytes, and neutrophils to clear large particles (e.g., microorganisms and apoptotic cells). In contrast, pinocytosis can occur in almost any cell through four distinct mechanisms: clathrin-dependent endocytosis, caveolae-dependent endocytosis, macropinocytosis, and clathrin/caveolae-independent endocytosis.

**Fig. 6:**
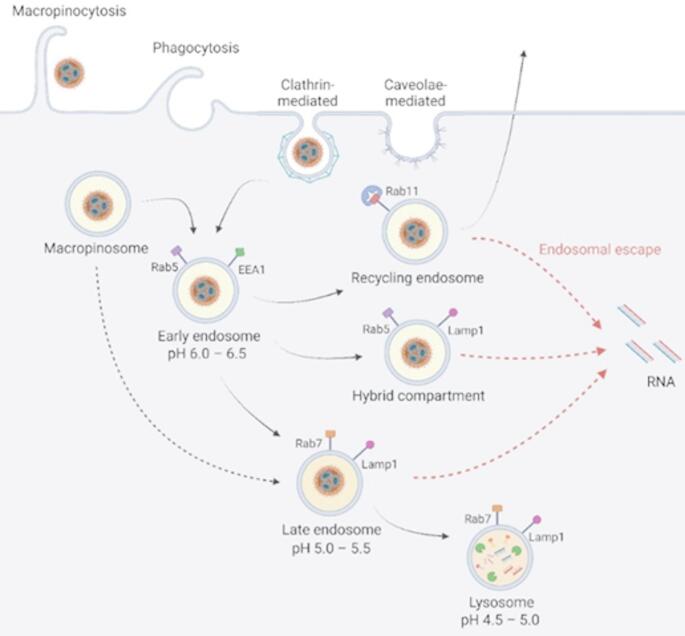
Schematic representation of RNA-LNP uptake, intracellular trafficking, and endosomal escape: Clathrin-mediated endocytosis and macropinocytosis were illustrated. Endosomal trafficking through the cell can be tracked by the Rab family of proteins, including Rab5, Rab7, and Rab11, which are associated with early, late, and recycling endosomes, respectively. Endosomal escape has been reported from different endocytic compartments in different studies. Adapted from [139]

Clathrin-mediated endocytosis is the predominant route for nucleic acid-loaded lipid-based nanoparticles, leading to lysosomal degradation unless endosomal escape occurs. Caveolae-mediated uptake provides an alternative pathway, allowing transport to intracellular compartments such as the Golgi and endoplasmic reticulum. LNPs may also enter cells via macropinocytosis, characterized by membrane ruffling and large vesicle formation that either fuse with lysosomes or recycle back to the membrane. In addition to these established routes, alternative pathways may occur, though their underlying mechanisms remain to be discovered [140]. Clathrin-mediated endocytosis (Fig. 6) begins with the invasion of the plasma membrane to create an endocytic vesicle. This vesicle then fuses with a sorting endosome. Its internal pH decreases as it matures due to the action of membrane-bound ATP-driven proton pumps [141] from the pH of 6.0-6.5 in early endosomes to the pH of 5.0-5.5 in late endosomes, and finally to the pH of 4.5-5.0 in lysosomes [137].

To avoid degradation of RNA in the hydrolytic lysosomal environment and achieve successful gene silencing effects, RNA needs to be delivered to the cytoplasm. If endosomal escape does not occur, the RNA-LNP system is either excreted from cells or degraded in hydrolase-rich lysosomes [137]. LNPs provide efficient endosomal escape due to the peculiar properties of the ionizable lipids. Indeed, at the acidic pH of endosomal vesicles, the positively charged LNP lipids interact with the negatively charged endosomal lipids, creating a cone-shaped geometry that causes the phospholipid bilayer structures to convert into hexagonal H_II_ phases, destabilizing the endosomal membrane and leading to the release of siRNAs into the cytoplasm [138]. Being neutral at the physiological pH, these lipids help to reduce the cytotoxicity of LNPs and their clearance by the fixed and free macrophages [142]. In addition, the gene-silencing potency of LNPs is highly dependent on the pK_a_ values of ionizable cationic lipids [142]. A study by Jayaraman et al. showed a strong bell-shaped curve correlation between the apparent pK_a_ of LNPs and their potency, with an optimal pK_a_ range of 6.2–6.5. From a library of ionizable lipids consisting of a dilinoleyl-based hydrophobic tail and various head groups, the authors distinguished the ionizable lipid (6Z,9Z,28Z,31Z)-heptatriaconta-6,9,28,31-tetraen-19-yl 4-(dimethylamino)butanoate (DLin-MC3-DMA) with an apparent pKa of 6.44 [143]. Patisiran (Alnylam Pharmaceuticals), the first siRNA therapeutic approved by the United States Food and Drug Administration (FDA) and the European Medicines Agency (EMA) in 2018 [12], employs the ionizable lipid DLin-MC3-DMA [5, 144]. Together with DLin-MC3-DMA, FDA-approved ionizable lipids such as SM-102 and ALC-0315 (used in the formulations of Moderna and Pfizer-BioNTech’s mRNA-LNP vaccines, respectively [145]) provide a solid foundation for further LNP advancements in the context of RNA delivery [146]. Various ionizable lipids have been developed to translate clinically siRNA-LNP formulations, including unsaturated, multi-tail, polymeric, biodegradable, and branched-tail ionizable lipids [138]. Moreover, ionizable lipid-like materials, such as C12-200, have been successfully used to prepare LNP-based siRNA systems [147–149]. Optimization studies of ionizable lipids have significantly improved the gene-silencing efficiency of LNPs [142]. Studies have demonstrated that only a tiny proportion (< 3.5%) of internalized RNA undergoes endosomal escape and reaches the cytoplasm following LNP-mediated delivery, while the majority is either degraded in lysosomes or recycled out of the cell [150, 151]. Using siRNAs labeled with gold nanoparticles, the endosomal escape was shown to be inefficient ($\:\sim$1‒2%) even for the optimized LNP formulations [150]. Endosomal escape is considered the major bottleneck for LNP-based therapeutics, and its better understanding is essential for developing more efficient siRNA-LNP systems [139].

Despite their effectiveness for cytoplasmic release, ionizable lipids have been shown to stimulate the immune system. It was shown that the amine headgroups in ionizable lipids stimulate immune responses to lipid nanoparticles by binding to receptors such as TLR4 and CD1d and promoting lipid-raft formation [152]. Recent studies have demonstrated their propensity to activate transcription factors, such as Nuclear Factor-kappa B (NF-κB) and Interferon Regulatory Factor (IRF) through TLR4 signaling [153, 154]. These findings underscore the pivotal role of ionizable lipid components in mediating inflammatory responses. These inflammatory responses likely contribute to the side effects observed with RNA therapeutics involving LNPs but may also enhance RNA vaccine efficacy [155].

#### Helper lipids

In addition to ionizable lipids, helper lipids are needed to improve the stability as well as the encapsulation and delivery efficiencies of LNPs. The term ‘helper lipid’ encompasses a variety of lipid types, including phospholipids, sterols, and PEG-lipids [16, 156]. Distearolyphosphatidycholine (DSPC) and 1,2-dioleoyl-sn-glycero-3-phosphoethanolamine (DOPE) are the most commonly used helper phospholipids in LNP formulations [16]. Phospholipids with a conical geometry (e.g., DOPE) enhance the LNP-mediated intracellular delivery of nucleic acids by promoting the formation of hexagonal lipid phases; however, they result in low stability due to the increased tendency for bilayer fusion and higher interaction with plasma proteins. On the other hand, saturated phosphatidylcholines with their cylindrical shape (e.g., DSPC) provide higher stability and longer in vivo half-life for LNPs thanks to their high gel-to-liquid phase transition temperatures (T_m_) [156]. Saturated helper lipids are better suited for delivering short siRNAs [110], while unsaturated lipids are more compatible with transporting longer mRNAs [147].

#### Cholesterol

Along with helper phospholipids, cholesterol (CHOL) is widely used in LNP formulations. The incorporation of CHOL between phospholipids enhances the stability of the lipid bilayer and significantly decreases the adsorption of serum proteins to the LNP surface [156]. The effect of CHOL on membrane fluidity changes depending on the situation. It increases membrane rigidity when inserted between phospholipids with low T_m_ and enhances membrane fluidity when inserted between phospholipids with high T_m_ [16]. CHOL also facilitates membrane fusion [15, 147, 157]. Interestingly, the substitution of CHOL with its analog β-sitosterol enhances fusion with the endosomal membrane, leading to increased transfection efficiency [158].

#### PEG-lipids

Finally, PEG-lipids play a crucial role in modulating the transfection efficiency and stability of LNP formulations. These lipids also affect the size and polydispersity of nanocarriers, encapsulation efficiency, biodistribution, and immune response [16]. PEG-lipids on the nanoparticle surface provide a hydrophilic steric barrier for the nanocarrier, avoiding particle aggregations and resulting in more homogeneous populations [16]. It has been observed that formulations without or with very low amounts of PEG-lipids lead to unstable and large LNPs (hydrodynamic diameter > 200 nm) with a polydisperse size distribution [159]. In contrast, using PEG-lipids as low as 0.5 mol% leads to stable, homogeneous, and small nanoparticles ($\:\sim$60 nm) [160]. Numerous studies have also shown that the nanoparticle size decreases as the molar percentage of PEG-lipids is increased in the range of 0.5–5 mol% [147, 160, 161]. Moreover, the hydrophilic steric hindrance provided by PEG-lipids prevents the attachment of plasma proteins (e.g., opsonins) to the LNP surface, reducing their capture by the MPS [20, 162]. PEG-lipids are required for higher stability and longer circulation times; however, their presence reduces cellular uptake efficiency or endosomal escape processes [163–165]. Typically, a PEG lipid content of ≤ 2 mol% is sufficient to achieve stealth characteristics without compromising efficient nanoparticle uptake by target cells [13]. Both the PEG chain and the anchoring lipid significantly influence the in vivo behavior of LNPs [166]. PEG2000, with a molecular weight of 2000 g/mol (~45 repetition units), is frequently used as it optimally balances extended circulation time and efficient gene delivery [164, 166, 167]. In contrast, shorter PEGs (≤ PEG1000) are ineffective in preventing protein corona formation and extending systemic circulation, and longer PEGs (≥ PEG5000) can significantly hinder cellular uptake and endosomal escape [166]. Regarding the lipid anchor, optimization studies have led to the development of diffusible PEG-lipids with C14 alkyl chains, which improve transfection efficiency thanks to their ability to rapidly detach from the LNP in the presence of a lipid sink [135]. In blood circulation, these lipids spontaneously transfer from LNPs to acceptor sites such as plasma lipoproteins and erythrocytes [162]. For example, DMG-PEG (C14) has been demonstrated to diffuse from LNPs in blood circulation quickly, enabling apolipoprotein E (ApoE) binding to the surface and subsequently leading to effective hepatocyte targeting [16, 168]. The desorption rate of a PEG-lipid from LNP decreases as its hydrophobic alkyl chain length increases [16, 162]. PEGs with longer lipid chains, such as DSPE-PEG (C18), can prolong blood circulation, potentially increasing LNP accumulation in extrahepatic organs and tumors [16, 166]. The type of PEG-lipid significantly impacts the LNP behavior and, therefore, should be selected based on the therapeutic target and the administration route. Some examples of PEG-lipids used in clinically approved LNP formulations include PEG-c-DMG (Patisiran, Alnylam Pharmaceuticals), PEG-DMG (mRNA-1273, Moderna) and ALC-0159 (BNT162b2, Pfizer-BioNTech) [16].

### Key aspects in reprogramming macrophages with RNA-LNPs

The therapeutic outcome of reprogramming macrophages using RNA-LNPs depends on several factors, such as the administration route, the targeting specificity, and the efficacy of RNA interference delivery. This section examines three critical aspects: local vs. systemic delivery approaches, passive vs. active targeting, and strategies to improve intracellular RNA interference delivery efficiency.

#### Local vs. systemic delivery approaches

Local administration enables precise targeting of macrophages at specific sites, such as local tumors. This method circumvents the majority of barriers encountered in systemic administration and could reduce systemic side effects and enhance therapeutic outcomes by increasing RNA interference concentrations at the tumor site. The efficacy of intratumorally administered nanoparticles is determined by several factors, including uniform dispersion throughout the tumor, an extended residence time, and internalization by target cells, all of which are influenced by nanoparticle characteristics [169]. Depending on the tumor size, the dose and injection rate must be tightly controlled to prevent leakage from the tumor and off-target effects. Although promising, this technique is invasive and necessitates professional injection skills [140, 169, 170]. The most critical barrier to its application is the difficulty of reaching deep tumor tissues because of their limited accessibility, requiring long needles or open surgeries. Furthermore, the use of long needles could harm healthy organs [169]. The number of studies using intratumoral (IT) administration of nanoparticles has been expanding in the last decade [169]. Most clinical trials on LNPs for cancer treatment have employed local delivery, primarily through IT injection [16]. However, this approach is inadequate for treating tumors with metastases or hematologic cancers [13].

**Fig. 7:**
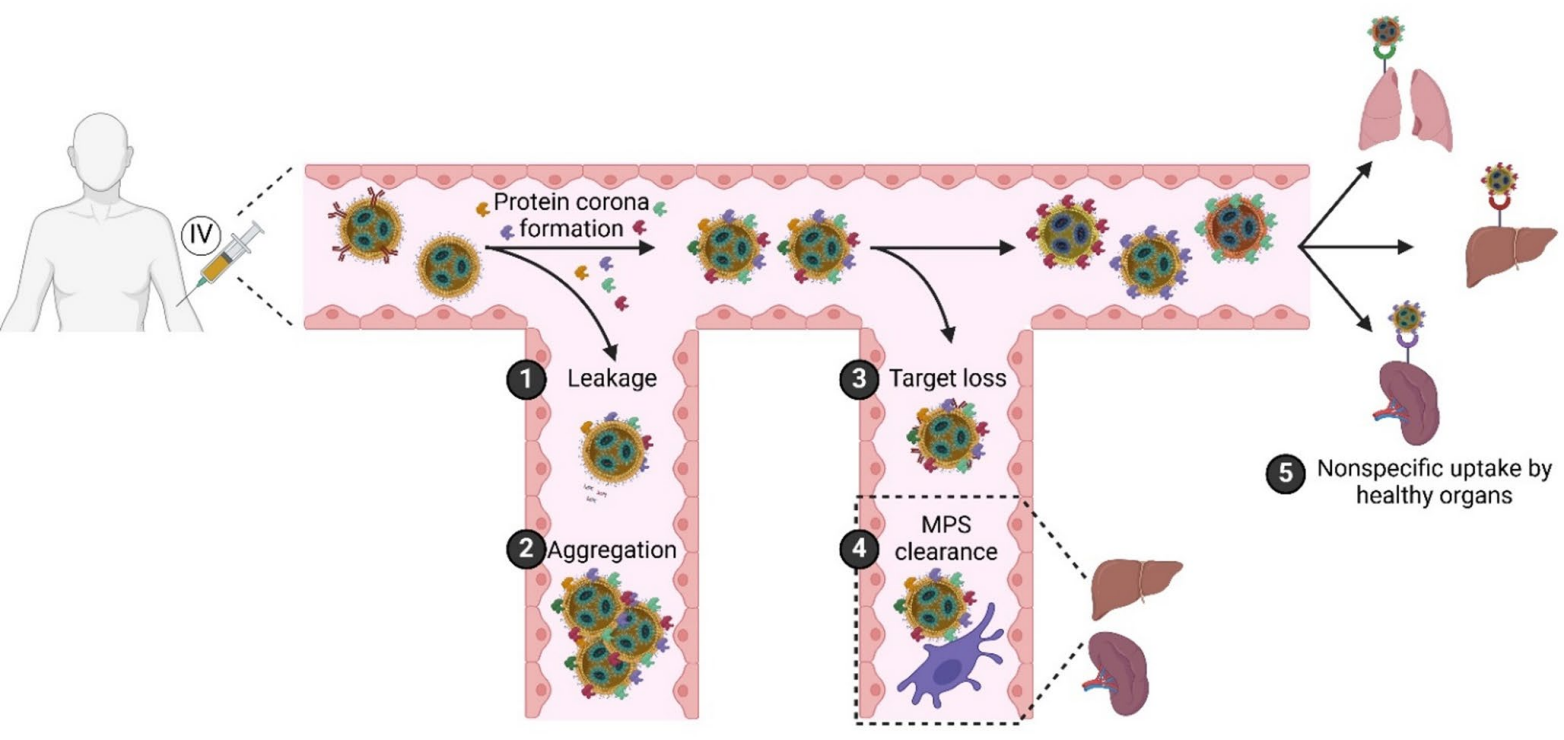
Challenges associated with systemic delivery of RNA interference-LNPs: Formation of protein corona on the LNP surface could cause (1) RNA interference leakage, (2) aggregation of LNPs, (3) lost targeting ability, (4) clearance by the MPS, or (5) nonspecific uptake by healthy organs. Adapted from [140]

On the other hand, systemic delivery refers to administering RNA interference-LNPs into the bloodstream, typically via intravenous (IV) injection. This approach is essential in metastatic cancers, as it allows for the simultaneous targeting of macrophages in multiple organs or tissues. Nevertheless, it encounters obstacles such as rapid clearance by the MPS, nonspecific uptake by healthy organs, and off-target effects (Fig. 7) [140]. Following systemic administration, the LNP surface is rapidly coated with plasma proteins (e.g., albumin, immunoglobulins, complement proteins, and apolipoproteins), forming a protein corona. This process, known as opsonization, enhances the elimination of nanoparticles by phagocytic cells [171]. Once administered intravenously, RNA-LNPs demonstrate a preferential accumulation in the liver and spleen, which can be attributed to the filtering functions of these organs and the presence of macrophages that phagocytose foreign particles [20, 140]. A 2022 review by Albertsen et al. identified 40 clinical trials investigating LNPs, of which 22 used local administration, such as intramuscular injection for vaccines and intratumoral delivery for cancer. Among the remaining trials, 11 employed intravenous administration, 7 for liver targeting, and 4 for cancer treatment [16]. The distribution of these trials highlights the difficulty of achieving effective non-liver targeting through systemic delivery routes. Therefore, attaining precise RNA interference-LNP delivery to extrahepatic targets requires optimizing targeting strategies, which will be examined in the following section.

#### Passive, active, and endogenous targeting

**Fig. 8:**
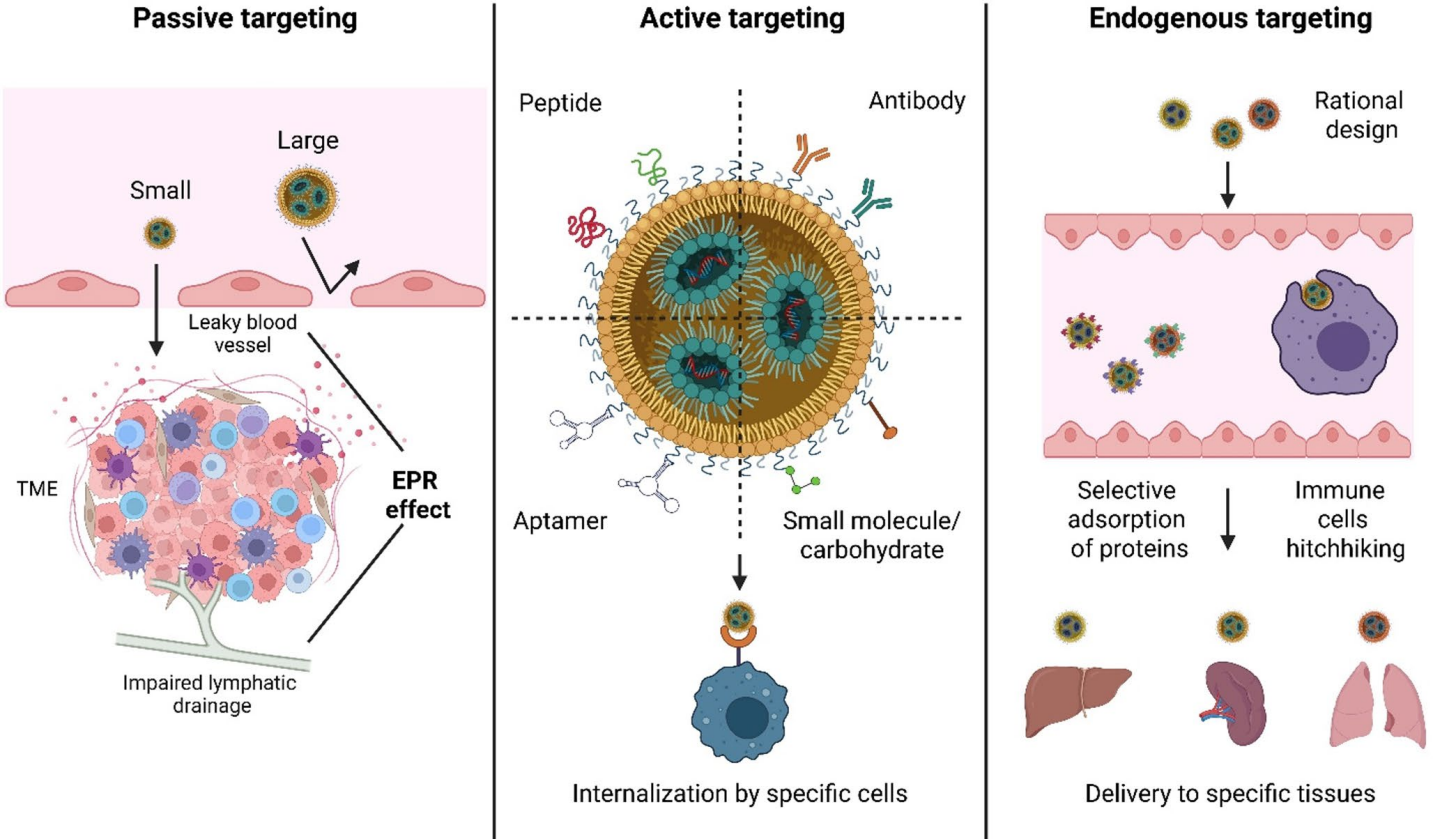
Targeting strategies of siRNA-LNPs. (**a**) Passive targeting relies primarily on LNP size and tumor-associated alterations in the tissue environment. (**b**) Active targeting utilizes ligands targeting cell-specific receptors. (**c**) Endogenous targeting depends on the protein corona that directs LNPs to specific sites in the body through receptor interaction or in situ cellular hitchhiking by immune cells that deliver the LNPs to the tumor. Adapted from [140]

Targeting TAMs for drug delivery holds great promise for cancer treatment. Main strategies include passive, active, and endogenous targeting (Fig. 8). These mechanisms can be employed individually or in combination to govern the biodistribution of LNPs [172].

Passive targeting involves controlling the properties of nanoparticles to take advantage of certain natural anatomical or physiological features or a pathophysiological condition [172]. For example, TAM targeting benefits from the natural physiology of macrophages and tumor-associated alterations in the tissue environment, and, as they are professional phagocytes, they naturally tend to internalize LNPs via phagocytosis [140, 173]. Another passive targeting strategy is to use particles with hydrophilic coatings (e.g., PEG) that typically exhibit prolonged circulation times and evade the MPS clearance [140, 171, 174], thereby enhancing the probability of passive uptake by tumor sites. Nevertheless, PEGylation may also restrain cellular uptake or the endosomal escape process [140]. Leaky vasculature and poor lymphatic drainage of tumors allow for passive targeting of macrophages within the TME via a phenomenon known as the enhanced permeability and retention (EPR) effect [175]. Doxil^®^, a PEGylated liposomal delivery system encapsulating doxorubicin currently in clinical use, employs passive targeting of tumors due to the EPR effect [173]. It has been reported that nanoparticles with a size smaller than that of the fenestrations in tumor vessels (200–800 nm) [140, 173, 175] are capable of permeating the TME, with the optimal size range being approximately 20–200 nm [173]. Furthermore, smaller LNPs can more effectively infiltrate stromal-rich tumor tissues, enhancing tumor accumulation [140]. Nevertheless, it should be noted that the EPR effect is less effective in poorly vascularized tumors or those with dense stromal barriers [13]. In brief, passive targeting is limited by non-specific cellular uptake. It lacks the specificity necessary for effective TAM delivery, requiring the development of advanced targeting strategies.

Active targeting is based on targeting moieties that selectively guide nanocarriers to the targeted cells and increase therapeutic efficacy while reducing off-target effects [126]. In this approach, the surface of nanocarriers is decorated with molecules (e.g., antibodies, aptamers, peptides, or small molecules) [140] specific to the receptor on macrophages. The endocytic uptake of actively targeted LNPs by macrophages occurs through specific receptor-ligand interactions involving mannose receptors, scavenger receptors, and Fc receptors [176]. It has been demonstrated that nanoparticles targeted to Fc and mannose receptors exhibit a notably shorter time to be internalized compared to those targeted to scavenger receptors, indicating the critical role of Fc and mannose receptors in the efficient uptake of nanoparticles [176]. In the context of cancer, the precise targeting of M2 TAMs is essential, and significant efforts have been made to develop strategies for this purpose. The mannose receptor CD206 is frequently used to target anti-inflammatory M2 macrophages due to its high expression on these cells [98]. Consequently, numerous studies have explored mannose-functionalized nanoparticles for actively delivering therapeutics to M2-like macrophages [97, 98, 131, 177, 178], demonstrating the efficacy of CD206-mediated uptake. Sialic acid-decorated nanocarriers have also shown potential for targeting M2 macrophages through specific interactions with CD169 (Siglec-1), a receptor highly expressed in these cells [99]. Other molecules, including folate [133] and anti-CD206 antibodies [179], have proven highly effective in targeting M2 TAMs. However, these targeting ligands also significantly bind to tissue-resident macrophages in healthy organs, dendritic cells, and epithelial cells [101, 180]. To specifically target M2 TAMs while avoiding non-specific binding to other leukocytes, some researchers have identified a peptide sequence (M2-targeting peptide, M2pep). Following the decoration of the nanoparticle surface with the M2pep, several authors demonstrated increased efficacy in delivering specific inhibitors and RNA interference to turn M2 macrophages into the immunostimulatory phenotype, M1 subtype [6, 101, 125, 180]. Kuninty et al. [181] conducted an interesting study targeting M2 macrophages. They designed liposomes containing peroxidized phospholipids, which are recognized and internalized by scavenger receptors on M2 macrophages. Delivering a STAT6 inhibitor (AS1517499), zoledronic acid, or cell wall lipopeptides to these cells inhibited the premetastatic microenvironment and tumor growth [181].

In addition to active and passive approaches, endogenous targeting emerges as a novel paradigm depending on a better understanding of how the protein corona regulates nanoparticle delivery. In this strategy, the nanoparticle is designed to interact with a particular subgroup of plasma proteins after intravenous administration, triggering internalization by specific cells [172]. The physicochemical properties of LNP formulations, together with patient-specific parameters such as sex and age, determine the composition of the proteins adsorbed. The protein corona governs the interaction of LNPs with macrophages [20, 140]. For instance, the coating of the LNP surface with complement 3 (C3) increased splenic distribution through complement receptors on macrophages in the spleen [182]. In another study, the protein corona significantly elevated the internalization of nanoparticles by M2 macrophages, which can be linked to the overexpression of phagocytic surface receptors against proteins adsorbed on nanoparticles [183]. The polarization state of macrophages plays a vital role in nanoparticle uptake [184–186], which could affect the pharmacokinetics and pharmacodynamics of the drug delivery system. An endogenous targeting mechanism, unrelated to macrophage-directed delivery, has been successfully utilized in Patisiran (Alnylam Pharmaceuticals). It involves the adsorption of ApoE onto the LNP surface, enabling recognition by low-density lipoprotein receptors (LDLRs) highly expressed on hepatocytes, thereby promoting selective liver accumulation [16, 168]. Another example, though again unrelated to macrophages, revealed an endogenous targeting strategy in solid tumors via the use of LNPs engineered to recruit vitronectin to their protein corona, contributing to αvβ3 integrin-mediated delivery to endothelial cells lining tumor blood vessels and enhancing siRNA transfection in tumor cells [187].

Another endogenous mechanism involves in situ cellular hitchhiking of nanoparticles that can lead to their accumulation in disease sites [188, 189]. Nanoparticles can be engineered to be internalized by blood circulating immune cells, such as monocytes and neutrophils, which naturally extravasate and infiltrate tumor tissues or metastatic sites, carrying the nanoparticles directly to the desired location [190]. This process is particularly effective for reaching metastatic lymph nodes or areas of inflammation associated with tumors. For example, this process has been described for cyclic RGD-decorated lipid nanoparticles in tumor mouse models [191]. In the context of macrophage reprogramming, Kuang et al. [192] designed lipid-based nanoparticles that target circulating monocytes and hitchhike with them to the central site of a glioblastoma in a mouse model [192]. The DOX·HCl contained within these nanoparticles is then released, inducing immunogenic cell death and the release of calreticulin, which contributes to macrophage reprogramming, dendritic cell maturation, and T cell activation [192]. Despite the preliminary success of hitchhiking strategy, efforts should focus on enhancing nanomaterials’ targeting efficiency and specificity to circulatory cells to enable their effective hitchhiking and delivery to specific tissues or cells. Further research is needed to optimize the selection and design of ligands or functional groups on the surface of nanomaterials to ensure efficient recognition and binding to the desired target. Premature release of loaded drugs during hitchhiking can impair the migration, homing, and other functions of hitchhiked cells or proteins, resulting in unpredictable therapeutic efficacy.

Ultimately, each of the three targeting strategies described in Fig. 8 —passive, active, and endogenous— could play a role in optimizing macrophage-directed drug delivery.

### Improving intracellular delivery efficiency

**Fig. 9:**
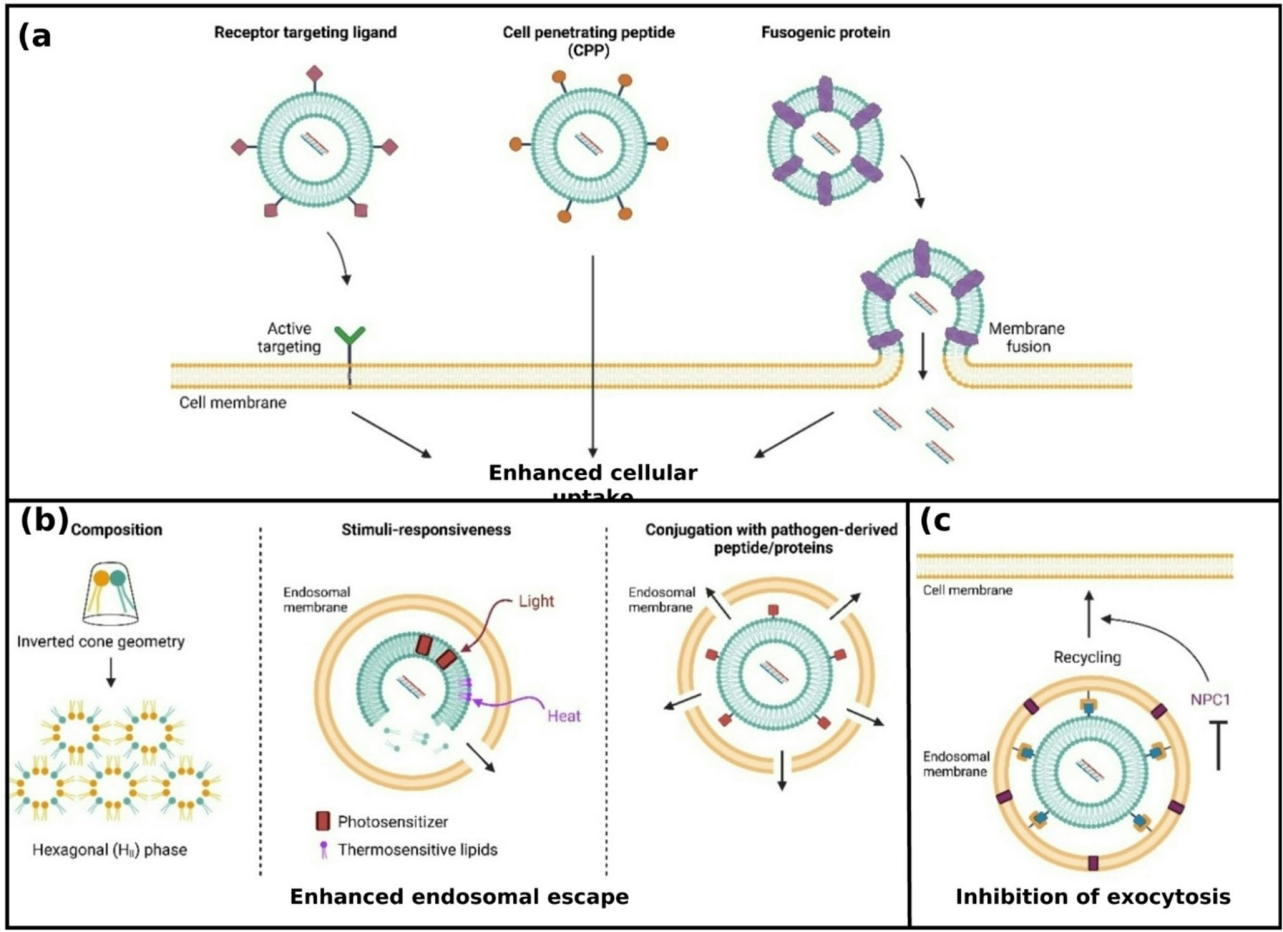
Strategies to improve intracellular delivery efficiency of RNA-LNPs. (**a**) Different strategies to enhance cellular uptake: modification of LNPs with receptor targeting ligands, cell penetrating peptides (CPPs), or fusogenic proteins. (**b**) Different strategies to improve endosomal escape: incorporation of inverted cone-shaped lipids into formulation and modification of LNPs with thermo/photosensitive molecules, or pathogen-derived peptides/proteins. (**c**) An example strategy to reduce exocytosis is the inhibition of NPC1. Adapted from [140]

Transfection efficiency is significantly influenced by LNP composition. For instance, PEG-lipids can hinder interactions between LNPs and cellular membranes, necessitating careful optimization of the PEG chain, anchoring lipid, and PEG-lipid molar ratio in the formulations [140]. A strategy to overcome this problem includes the development of pH-sensitive PEG-lipids, which remain stable under physiological conditions while detaching from the surface of LNPs in acidic microenvironments. This approach retains PEG’s stealth properties while reducing its adverse effects on transfection efficiency. In the study of Xiao et al. [6], researchers designed micelleplexes functionalized with M2pep, where these peptides were embedded within a pH-sensitive PEG corona. The cleavage of the surface-bound PEG-lipids in the acidic TME (pH$\:\sim$6.5–6.8) enables the precise delivery of therapeutic agents to M2 TAMs. As seen in this example, one strategy to enhance cellular uptake is to induce endocytosis. This can be achieved by surface modification of LNPs by conjugating receptor-targeting ligands or cell-penetrating peptides (CPPs) (Fig. 9a) [140]. Modifying LNPs with the RGD peptide (Arg-Gly-Asp)-based ionizable lipids [193], the macrophage-targeting peptide CRV (sequence CRVLRSGSC) [146], or the protamine-derived CPP [194] has been shown to facilitate nanoparticle internalization. While enhancing endocytic uptake could improve cytoplasmic availability, non-endocytic delivery approaches offer a direct route to the cytoplasm and avoid endosomal entrapment. An interesting strategy consists of mimicking viral mechanisms of cell entry via membrane fusion, allowing the bypass of endocytic pathways to enhance cytoplasmic delivery (Fig. 9a). Entos Pharmaceuticals^®^ engineered a fusogenic proteolipid vehicle consisting of neutral lipids and fusion-associated small transmembrane proteins, which enables the direct delivery of nucleic acids into the cytoplasm [195].

Although LNP endocytosis is effective, obstacles such as lysosomal degradation and poor RNA bioavailability in the cytoplasm highlight the necessity to enhance endosomal escape to obtain better therapeutic outcomes. The mechanisms underlying endosomal escape have evolved significantly over the past few decades. In the late 1990s, the proton sponge hypothesis emerged to explain nanoparticles containing cationic polymers. This model proposes that the cationic polymers buffer the acidic endosomal environment, causing osmotic swelling and subsequent membrane rupture [196]. In this context, the research by our group has significantly contributed to developing liposome-protamine systems for siRNA delivery [197]. Next, researchers identified an alternative mechanism involving cationic lipids (e.g., DOTAP) in LNP formulations. These cationic lipids interact with negatively charged endosomal membrane lipids via electrostatic interactions, destabilizing the bilayer structure and facilitating cargo release [198, 199]. In the 2000s, multiple mechanisms have been proposed to explain how endosomal escape occurs, including the flip-flop, membrane fusion, destabilization, and pore formation models [140]. While positively charged lipid-based nanoparticles typically demonstrate a higher cellular uptake, they are also associated with a higher risk of cytotoxicity and increased opsonization by anionic serum proteins [200]. A breakthrough in LNP technology came with the introduction of ionizable cationic lipids, which remain neutral at physiological pH but become positively charged in the acidic environment of endosomes [138, 201]. This pH-responsive behavior enables escape mainly via the flip-flop mechanism, where lipid rearrangement induces the formation of non-bilayer hexagonal phases, promoting membrane fusion and cargo release [140]. This advancement was pivotal in developing siRNA- and mRNA-based LNP therapies, including coronavirus disease 2019 (COVID-19) vaccines [15].

The endosomal escape process is challenging to investigate due to its transient nature and short-lived duration. Even though it is widely accepted that nucleic acid cargos are released into the cytoplasm before fusing with the lysosome, the specific sites where this release occurs continue to be debated [140]. Various studies are reporting that LNPs release the siRNA cargo in early endosomes [150], late endosomes [151], or hybrid compartments having the properties of early and late endosomes [150, 151]. This underscores the impact of LNPs’ physicochemical properties and different cell types on the endosomal escape process. Regarding the structure of lipids in LNPs, the ionizable lipids can be engineered to mimic inverted-cone phospholipids to trigger the formation of hexagonal phases, leading to efficient cargo escape. Another strategy includes the introduction of unsaturated bonds or biodegradable groups into ionizable lipids [138]. Moreover, incorporating unsaturated phosphatidylethanolamines, such as DOPE, into the formulation increases the endosomal escape efficiency by inducing hexagonal membrane transformations (Fig. 9b) [156]. Similarly, modifications to CHOL or its substitution with analogs can be employed as alternative strategies to optimize endosomal escape. For example, replacing CHOL with plant-derived β-sitosterol has improved the transfection efficiency of LNPs, potentially thanks to its ability to facilitate enhanced fusion with the endosome [158, 202]. Furthermore, modifying LNPs with pathogen-derived peptides or proteins (e.g., HIV-1 TAT, PV L2, HA2, and CADY) represents another strategy to facilitate endosomal escape through membrane destabilization, fusion, or pore formation (Fig. 9b) [140]. Stimuli-responsive LNPs (e.g., thermoresponsive, photoresponsive) have also been investigated to enhance endosomal escape (Fig. 9b) [203, 204].

Interestingly, limiting exocytosis also represents an alternative strategy to increase the siRNA availability in the cytoplasm [140]. Altering the LNP composition, such as using hydroxycholesterols instead of cholesterol [202], or inhibiting a critical modulator of the exocytosis pathway, such as Niemann-Pick type C1 (NPC1) (Fig. 9c) [149], has been demonstrated to improve the retention of lipid-based nanoparticles.

Despite considerable progress in recent years and ongoing efforts, the intracellular delivery efficiency of siRNA-LNP systems continues to pose a significant challenge. Addressing this limitation is crucial for harnessing their full therapeutic potential.

### Duration of the inhibitory effect of RNA interference

Achieving long-term therapeutic knockout effects often requires repeated dosing, which can sometimes limit its application. Ongoing research aims to further prolong this inhibition and to enable more effective and less frequent therapeutic interventions. Although naked siRNAs and miRNAs are rapidly degraded, LNP delivery can significantly extend their stability and gene-inhibiting effects, which generally range from days to several weeks depending on the formulation and application [205].

One in vitro study revealed that siRNA protected in solid lipid nanoparticles (SLNPs) retains functional activity after a six-day incubation at 37 °C [206]. Another study measured the duration of the inhibitory effect obtained using a hydrophobic ion pairing approach that employs the cationic lipid DOTAP to load siRNA into tristearin SLNPs [207]. Intradermal injection of these nanocarriers into the footpads of mice resulted in prolonged siRNA release over 10–13 days.

The liver-targeted Patisiran was administered once every 3 weeks during the Phase III trial [208]. However, few reports show how long the inhibitory effects last in cancer cells. So far, there has been significant variability in dosage frequency regarding Phase I cancer RNAi clinical trials, ranging from once to twice per week to twice a month [209–211].

Regarding the activity in macrophages, past studies on siRNA have shown that its dilution due to cell division may affect the duration of gene silencing [212]. The siRNA molecules can achieve target protein inhibition superior to 80% at nanomolar concentrations, and their enhanced intracellular stability enables knockdown that can last for weeks in nondividing cells [212]. Using polymer nanoparticles to deliver siRNA against TNF-α oversecreted in macrophages, the inhibition lasted at least one week [213]. Regarding miRNA and given these factors, it is more accurate to think of miRNA silencing in macrophages as a dynamic and continuous process that can last as long as the miRNA is present and active, and its target mRNA is being expressed. Like for siRNA, the strength and specificity of the silencing effect might fluctuate based on the cellular environment and all other factors listed above.

### Preclinical studies using siRNA or miRNA-LNPs

This section reviews selected preclinical studies published recently that utilize siRNA or miRNA-LNPs to reprogram the TME, yielding promising therapeutic outcomes. (Table 1). Shobaki et al. [5] developed siRNA-LNPs based on a pH-sensitive cationic lipid CL4H6 to silence STAT3 and HIF-1α in TAMs. Following intravenous administration, siRNA-loaded CL4H6-LNPs demonstrated a high uptake and efficient gene silencing in TAMs in a human tumor xenograft of renal cell carcinoma (OS-RC-2) model, triggering an anti-tumor therapeutic response. Notably, the treatment was associated with an increased infiltration of macrophages into the TME, an increased tendency toward M1 polarization, and a reduction in angiogenesis and tumor cell activation [5].

**Table 1:** Preclinical studies employing RNA interference-LNPs in cancer immunotherapy

Target		Composition	Targeting ligand	Cell/animal model	Administration route for in vivo studies	Effects	Ref
**siRNA**	siSTAT3 & siHIF-1α	CL4H6: CHOL: PEG- lipid (DMG-PEG2000 or DSG-PEG2000)	None	- Mice bearing human tumor xenograft of renal cell carcinoma (OS-RC-2)	IV	- Higher infiltration of macrophages into the TME and increase in the level of M1 macrophages - Reduced angiogenesis and tumor cell activation/invasiveness	[5]
	siMerTK	DLin-MC3-DMA: DSPC: CHOL: DMG-PEG2000	None	- IL-4-treated BMDMs - - Mice bearing CT26 and MC38 liver metastasis & Mice bearing CT26 peritoneal metastasis of colorectal cancer	IV (liver metastasis) IP (peritoneal metastasis)	- Increased level of M1 (iNOS) and decreased level of M2 (Arg1) markers in vitro - Reduced tumor growth and better survival in vivo	[214]
	siHO1	Lipid 8: DSPC: CHOL: DMG-PEG2000	Anti-PD-L1 antibody	- Ex vivo bone marrow cells - Mice bearing B16F10 melanoma	IV	- Increased levels of pro-inflammatory (TNF-α and IL12p40) and decreased levels of anti-inflammatory (IL-10) cytokines in ex vivo study - Enhanced sensitivity to chemotherapeutics and improved responsiveness to immune checkpoint inhibitor therapy - Reduced TAMs in the TME, higher infiltration of CD8 + cytotoxic T cells, and reduced tumor growth and prolonged survival (in combination with chemotherapy and an immune checkpoint inhibitor)	[215]
	siPDL1 & mOX40L	DLin-MC3-DMA: DOPE: CHOL: C16-PEG2000	None	- Mouse macrophage cell line, J774 - Mice bearing B16F10 melanoma	IT	- Increased level of M1-associated markers (CD80 and CD86) in vitro - Reduced tumor growth, higher infiltration of CD4 + and CD8 + cells in the TME, and immune activation within tumor-draining lymph nodes - Internalization by immune cells (CD45 + leukocytes) within the TME, indicating their potential to reprogram TAMs	[105]
	siDICER	Dlin-MC3 DMA: DMG-PEG: DSPC: CHOL DSPE-PEG-M2 pep	M2 targeting Peptide	-	IV	- Significant anti-tumor effect with an improved immune microenvironment - Reprogramming of M2-like macrophages through downregulation of miR-148a-3p and miR-1981-5p	[216]
**miRNA**	miRNA-199-5p miRNA-204-5p	DODAP: PEG2000-CER: DSPC: CHOL	None	- -In vitro: THP-1 differentiated - In vivo: A375 melanoma and M14 melanoma mouse model	IV	- Reduced melanoma cell growth - Restricted recruitment and reprogramming of pro-tumoral macrophages	[217]
	miRNA-155	EDOPC: DC-CHOL: DPHPE: DSPE-PEG	Anti-SIRPα antibody	- In vitro: BMDM and B16F10 cells - In vivo: B16F10 melanoma mouse model	IT	- Reprogramming of TAMs - Activation of T cells’ phagocytic activity against tumor cells through the blockade of CD47-SIRPα interaction	[218]

In the study by Zhou et al. [214], the researchers synthesized siRNA-LNPs targeting the MerTK receptor on TAMs, which play a role in clearing apoptotic cells. They showed that inhibiting MerTK results in the accumulation of apoptotic cells within the TME, which triggers a pro-inflammatory response. Their treatment enhanced antitumor immunity in both liver and peritoneal metastasis models of colorectal cancer. While focusing on inhibiting MerTK-mediated efferocytosis, the results suggest a functional reprogramming of TAMs toward the M1 phenotype. This is evidenced by increased and decreased classical M1 and M2 markers in the IL-4-treated BMDM cellular model.

Furthermore, Yong et al. [215] developed anti-PD-L1 antibody-functionalized LNPs loaded with siRNA against heme oxygenase-1 (HO1) to simultaneously target cancer cells and tumor myeloid cells, aiming to achieve a dual effect of enhanced chemotherapeutic efficacy and immunological reprogramming. The findings demonstrated that HO1 inhibition sensitizes tumor cells to conventional chemotherapeutics and boosts the immune response by reprogramming tumor myeloid cells. Following LNP treatment with doxorubicin, a notable reduction in total myeloid cells and M2-like TAMs in the tumor was observed in B16F10 melanoma-bearing mice. Further investigation on ex vivo bone marrow cells indicated that the treatment inhibits the differentiation of monocytes into TAMs and increases the level of pro-inflammatory cytokines, including TNF-α and IL12p40, while decreasing the anti-inflammatory cytokine IL-10.

In another study, Walters et al. [105] successfully formulated LNPs that encapsulate a combination of siRNA against PD-L1 and mRNA encoding OX40L. The objective was to silence and stimulate the expression of immunosuppressive and immunostimulatory immune checkpoint targets, respectively, for reprogramming the TME towards a more immunostimulatory state. The researchers showed that these SNALPs increased the M1-associated markers CD80 and CD86 in a mouse macrophage cell line, J774. Subsequent intratumoral administration in mice bearing B16F10 melanoma has resulted in a higher level of CD4 + and CD8 + infiltrates in the tumor and a reduction in tumor growth. While not directly related to TAMs, the internalization of LNPs by immune cells (CD45 + leukocytes) within the TME suggests that they have the potential to be developed for reprogramming TAMs in vivo.

Xia et al. [216] delivered an LNP-entrapped siRNA targeting DICER1, a mutation highly expressed in TAMs and closely associated with M2 polarization in colorectal cancer liver metastasis. After intravenous administration, the siRNA exerted a significant anti-tumor effect with an improved immune microenvironment in a colorectal cancer liver metastasis mouse model. Macrophage depletion experiments further suggested that this effect largely depended on the presence of TAMs. Mechanistically, DICER inhibition reprogrammed M2-like macrophages by downregulating miR-148a-3p and miR-1981-5p [216].

There are two cases where LNPs were used to deliver miRNA for macrophage reprogramming in cancer. In the first one, LNPs co-encapsulating miRNA-199-5p and miRNA-204-5p, combined with target therapy, inhibited tumor growth after intravenous administration in a melanoma mouse model and blocked the development of drug resistance. Mechanistically, they acted by directly reducing melanoma cell growth and indirectly hampering the recruitment and reprogramming of pro-tumoral macrophages [217]. In a recent study, lipid nanoparticles were modified with an anti-SIRPα antibody for the targeted delivery of miRNA-155 to TAMs to enhance their anti-tumoral phenotypes within the tumor microenvironment [218]. The SIRPα-specific binding of the LNPs to the surface of TAMs allowed their reprogrammation through the intracellular delivery of miR155. These reprogrammed TAMs increase the secretion of pro-inflammatory cytokines. In addition to TAM-specific miRNA delivery, the binding of SIRPα by the anti-SIRPα antibody blocks the interaction between CD47 on tumor cells and SIRPα on TAMs. This interruption of the CD47-SIRPα interaction activates the phagocytic activity of T cells against tumor cells [218]. Taken as a whole, all these fascinating results are too recent, and their clinical translation remains ongoing. However, the further advancement of these strategies should be eased by the availability of LNP formulations already on the market.

## Challenges and future directions

Although promising, there are several challenges associated with RNA interference-LNP-mediated macrophage reprogramming. This section will mainly focus on the delivery challenges. However, concerning target cells, one of the key hurdles is the incomplete understanding of macrophage heterogeneity. Macrophages exhibit diverse phenotypes and functions within different physiological and pathological contexts. While the M1/M2 polarization model provides a valuable framework, it oversimplifies the complex spectrum of macrophage states observed in vivo, especially in the dynamic TME. Context-dependent changes in expression characterize polarization, and it is essential to note that not all M1 or M2 markers necessarily upregulate or downregulate upon activation. Therefore, single-marker assessments may misclassify macrophages, highlighting the importance of employing a panel of M1 and M2 markers [219]. Moreover, emerging single-cell sequencing [220–224] and spatial transcriptomics [225–227] techniques may allow a more profound understanding of macrophage diversity in vivo. These advanced methods have already revealed distinct subpopulations of TAMs, but the molecular mechanisms governing their plasticity remain poorly understood [228].

In addition to the above, targeting specific tissues or cells beyond the liver using RNA-LNPs via IV administration represents another ongoing challenge [16, 229]. The need to target particular macrophage subsets within the TME makes it even more complex, where M1-like and M2-like phenotypes coexist. While targeting M2 TAMs, it should be considered that the drug delivery system has a minimal effect on the tissue-resident M2 macrophages in healthy organs [6] to prevent the activation of M1 macrophages in non-target tissues, which could lead to a systemic inflammatory reaction [29]. Considering all these challenges, it is unsurprising that local injection has been predominantly used over IV administration in clinical trials using RNA interference-LNPs [16]. However, intratumoral injection is unsuitable for treating metastatic or hematologic cancers [13]. Therefore, developing advanced targeting strategies (e.g., active or endogenous targeting) is needed to increase the therapeutic efficacy of RNA-LNPs while reducing the risk of side effects [6, 125]. Understanding and modulating the protein corona could improve nanoparticle biodistribution [140, 229]. Recent research has revealed that selective organ targeting (SORT) LNPs can achieve preferential accumulation in extrahepatic organs, such as the spleen and lungs. This is accomplished by manipulating nanoparticle surface interactions with serum proteins, ultimately guiding receptor-mediated uptake [229]. Also, optimizing surface modifications, such as PEGylation, is essential to prolong circulation time, minimize hepatic sequestration, and improve tumor accumulation [13]. Moreover, due to tumors’ highly complex immune microenvironment, macrophage-centered immunotherapies alone may not be potent enough to eliminate tumors [228], and the combination of TAM reprogramming with other therapeutic modalities (e.g., chemotherapy, radiotherapy, or immunotherapy) should be further explored [2, 46]. LNP-mediated co-delivery strategies, including multiple RNAs targeting distinct pathways or siRNA-mRNA pairs, also require further investigation [13, 105]. In addition, the typical characteristics of TME, such as acidity, abnormal vasculature, hypoxia, elevated interstitial pressure, and dense stromal network, could restrict the efficient delivery of RNA interference-LNPs. These complexities highlight the necessity for appropriate in vitro (e.g., 3D models) and in vivo models to evaluate the distribution of siRNA-LNPs [13]. Recent efforts have also explored various strategies, such as pH-sensitive nanoparticles [6], to exploit the TME-associated characteristics to enhance drug delivery to tumors. At the cellular level, there is a need to understand endosomal escape better in order to improve the amount of RNA reaching the cytoplasm.

Another critical research gap regarding RNA interference-LNPs is long-term safety concerns. LNPs pose immunogenicity and toxicity challenges, primarily due to ionizable cationic lipids and PEG-lipids [13]. The immunogenicity of ionizable cationic lipids has been primarily linked to the hydrophilic amine headgroup, which has been demonstrated to trigger both the innate and adaptive immune pathways by inducing lipid-raft formation and binding to the receptors TLR4 and CD1d [152]. A critical challenge, therefore, is to design new ionizable lipids that are more effective in endosomal escape and less immunogenic. Another major concern is the risk of complement activation-related pseudoallergy (CARPA), a hypersensitivity reaction caused by the interaction of PEGylated LNPs with the complement system. While PEGylation improves the pharmacokinetics of LNPs, repeated administration could elicit anti-PEG IgG and IgM responses, activating the complement cascade [140]. Therefore, a deeper investigation into the interplay between LNP components and immune system molecules is essential to enhance the safety profile of siRNA-LNPs [152]. Additionally, the binding of anti-PEG antibodies on PEGylated LNPs accelerates nanoparticle clearance by immune cells through Fc receptor-mediated pathways, reducing the efficacy of subsequent treatments. Thus, emerging strategies focus on replacing PEG with alternative molecules to reduce safety concerns while optimizing therapeutic efficacy [140]. Long-term off-target effects represent another key issue. The possibility of siRNA-LNP accumulation in non-target organs poses a risk of cumulative toxicity, inflammation, and disruption of normal tissue function [140]. Beyond off-target effects, while RNA interference-LNP-based macrophage reprogramming is promising, its stability over time is uncertain. There is a possibility that reprogrammed macrophages could revert to their tumor-promoting states due to ongoing signals from the TME. Thus, long-term studies are required to assess the durability of macrophage reprogramming.

## Conclusion

Macrophages can act as tumor supporters or fighters, and their plasticity makes them an attractive target for therapeutic reprogramming to treat cancer. siRNA and miRNA-LNPs represent a potent platform for regulating macrophage function thanks to their precise gene silencing capabilities nd the efficient delivery properties of LNPs. Recent advances in LNP composition and targeting strategies have greatly enhanced the therapeutic potential of this drug delivery system. However, several challenges remain. Issues related to macrophage complexity, in vivo targeting specificity and long-term safety concerns must be addressed for its clinical translation as a viable cancer immunotherapy strategy. Unlocking the full potential of RNA interference-LNP-based macrophage reprogramming will provide a transformative approach to treating cancer and a wide range of macrophage-related disorders, including chronic inflammation, infection, and autoimmune diseases.

## Abbreviations

ADA: Anti-drug antibody
ADCC: Antibody-dependent cellular cytotoxicity
ADCP: Antibody-dependent cellular phagocytosis
Ago2: Argonaute-2
ANG2: Angiopoietin-2
ApoE: Apolipoprotein E
Arg1: Arginase-1
BMDM: Bone marrow-derived macrophage
C3 (or 5): Complement component 3 (or 5)
CAR: Chimeric antigen receptor
CARPA: Complement activation-related pseudo allergy
Cas9: CRISPR-associated protein 9
CCL: C-C chemokine ligand
CCR: C–C chemokine receptor
CD: Cluster of differentiation
CD169: Sialic acid binding Ig-like lectin 1 (also known as Siglec-1)
CD206: Cluster of differentiation 206 (also known as mannose receptor)
cGAMP: Cyclic guanosine monophosphate–adenosine monophosphate
CHOL: Cholesterol
COVID-19: Coronavirus disease 2019
COX-2: Cyclooxygenase-2
CpG: Cytosine-phosphate-guanine
CPP: Cell-penetrating peptide
CRISPR: Clustered regularly interspaced short palindromic repeats
CSF-1: Colony-stimulating factor 1
CSF-1R: Colony-stimulating factor 1 receptor
CXCL: C–X–C motif chemokine ligand
DAMPs: Damage-associated molecular patterns
DCs: Dendritic cells
DLin-MC3-DMA: (6Z,9Z,28Z,31Z)-Heptatriaconta-6,9,28,31-tetraen-19-yl 4-(dimethylamino)butanoate
DMG: 1,2-dimyristoyl-sn-glycerol
DOPE: 1,2-dioleoyl-sn-glycero-3-phosphoethanolamine
DOTAP: 1,2-dioleoyl-3-trimethylammonium-propane
DSG: 1,2-distearoyl-sn-glycerol
DSPC: Distearolyphosphatidycholine
dsRNA: Double-stranded RNA
EC: European Commission
ECM: Extracellular matrix
EEA1: Early endosome antigen 1
EGF: Epidermal growth factor
EPR: Enhanced permeability and retention
FDA: United States Food and Drug Administration
FGF: Fibroblast growth factor
Fizz1: Found in inflammatory zone 1 (also known as Retnla)
GM-CSF: Granulocyte-macrophage colony-stimulating factor
HA2: Hemagglutinin 2
hATTR: Hereditary transthyretin amyloidosis
HIF: Hypoxia- inducible factor
HIV-1 TAT: Human immunodeficiency virus type 1 trans-activator of transcription
HO1: Heme oxygenase-1
IDO: Indoleamine 2,3-dioxygenase
IFN-γ: Interferon-gamma
Ig: Immunoglobulin
IKK: IκB kinase
IL: Interleukin
iNOS: Inducible nitric oxide synthase
IRF: Interferon regulatory factor
IT: Intratumoral
IV: Intravenous
IκBα: Inhibitor of nuclear factor kappa B alpha
JAK: Janus kinase
KLF4: Kruppel-like factor 4
Lamp1: Lysosomal-associated membrane protein 1
LDLR: Low-density lipoprotein receptor
LNP: Lipid nanoparticle
LPS: Lipopolysaccharide
M2pep: M2-targeting peptide
MARCO: Macrophage receptor with collagenous structure
MDSC: Myeloid-derived suppressor cell
MerTK: MER proto-oncogene tyrosine kinase
MHC II: Major histocompatibility complex II
miRNA: MicroRNA
MMP: Matrix metalloproteinase
MPS: Mononuclear phagocyte system
mRNA: Messenger RNA
NF-κB: Nuclear factor kappa B
NK cells: Natural killer cells
NO: Nitric oxide
NPC1: Niemann-Pick type C1
OX40L: OX40 ligand
PD-1: Programmed cell death protein 1
PDGF: Platelet-derived growth factor
PD-L1: Programmed cell death ligand 1
PEG: Polyethylene glycol
PI3K: Phosphoinositide 3 kinase
pK_a_: Acid dissociation constant
PO4: Phosphodiester
PPAR: Peroxisome proliferator-activated receptor gamma
PRR: Pattern recognition receptor
PS: Phosphorotioate
PV L2: L2 protein of papillomavirus
Rab: Ras-associated protein
RelA: Nuclear factor kappa-B p65 subunit (also known as p65)
RGD: Arginine-glycine-aspartic acid
RISC: RNA-induced silencing complex
RNAi: RNA interference
ROS: Reactive oxygen species
siRNA: Small interfering RNA
siRNA-LNP: SiRNA-loaded LNPs
SIRPα: Signal-regulatory protein alpha
SNALP: Stable nucleic acid-lipid nanoparticle
STAT: Signal transducer and activator of transcription
STING: Stimulator of interferon genes
TAMs: Tumor-associated macrophages
TGF-β: Transforming growth factor beta
TIE2: Tyrosine kinase with immunoglobulin and EGF homology domains 2
TIM-3: T-cell immunoglobulin and mucin-domain containing 3
TLR: Toll-like receptor
T_m_: Gel-to-liquid phase transition temperature
TME: Tumor microenvironment
TNF: Tumor necrosis factor
TNF-α: Tumor necrosis factor alpha
Treg: Regulatory T cell
VEGF: Vascular endothelial growth factor
VISTA: V-domain immunoglobulin suppressor of T-cell activation
Ym1: Chitinase-like protein 3 (also known as Chi3l3)
